# FedMamba-IoMT: Federated state space models with differential privacy and byzantine resilience for privacy-preserving intrusion detection in Internet of Medical Things

**DOI:** 10.1371/journal.pone.0355601

**Published:** 2026-08-10

**Authors:** Yasser Mohammad Al-Sharo, Mohammed Tawfik, Ali Mansour Almadani, Amr H. Abdelhaliem, Islam S. Fathi, Gaber Hassan

**Affiliations:** 1 Department of Cyber Security, Ajloun National University, Ajloun, Jordan; 2 Faculty of Computer and Information Technology, Sana’a University, Sana’a, Yemen; 3 Department of Cyber Security, Faculty of Science and Information Technology, Irbid National University, Irbid, Jordan; 4 Department of Computer Science, Faculty of Information Technology, Ajloun National University, Ajloun, Jordan; 5 Department of Computer Science, Faculty of Computers and Information, Arish University, Al-Arish, North Sinai, Egypt; Maulana Abul Kalam Azad University of Technology West Bengal, INDIA

## Abstract

The proliferation of Internet of Medical Things (IoMT) devices has created critical cybersecurity challenges demanding intrusion detection systems that achieve high accuracy across diverse attack taxonomies while preserving patient privacy across institutional boundaries. Existing federated learning (FL) approaches face an inherent tension: Transformer-based architectures achieve strong detection performance but incur quadratic computational complexity and substantial communication overhead, while lightweight classifiers sacrifice representational capacity. Moreover, most FL-based intrusion detection systems lack formal privacy guarantees and robustness against adversarial participants. This paper introduces FedMamba-IoMT, the first federated State Space Model framework for privacy-preserving intrusion detection in IoMT networks, incorporating differential privacy (DP-SGD), Byzantine-resilient aggregation, and multi-level explainability. The proposed architecture reformulates tabular network traffic features as pseudo-sequential tokens processed through stacked selective State Space Model (Mamba) blocks with gated residual connections, achieving linear computational complexity 𝒪(n) with 78% fewer parameters than Transformer alternatives. We design a novel FedMamba aggregation strategy that weights client contributions by a convex combination of dataset proportion and inverse validation loss, augmented with a cosine similarity-based Byzantine filter that detects and excludes malicious model updates. Integration of DP-SGD with Rényi differential privacy accounting provides formal privacy guarantees (ε∈{1.0,2.0,3.0,5.0,8.0}, $\delta =10^{-5}$) while maintaining competitive accuracy. Comprehensive evaluation across three benchmark datasets—Edge-IIoTset (2,219,201 samples, 15 classes), CICIoMT2024 (3,204,537 samples, 19 classes), and Gotham Dataset 2025 (496,191 samples, 8 high-level traffic categories)—demonstrates that FedMamba-IoMT achieves 99.47±0.04%, 99.52±0.04%, and 98.90±0.04% multiclass accuracy without DP, and 98.52%, 98.18%, and 97.16% at ε=3.0, surpassing all prior federated IDS approaches. Byzantine resilience experiments demonstrate that the proposed defense maintains >95% accuracy under 30% malicious clients across label-flipping, model poisoning, and free-rider attacks. Gradient inversion analysis confirms that FedMamba’s compact parameterization (135K parameters, 0.52 MB) provides 2× higher reconstruction error compared to Transformer-based FL, and the integrated SHAP and LIME explainability framework supports regulatory compliance with the FDA’s 2023 cybersecurity guidance for medical devices.

## Introduction

The Internet of Medical Things (IoMT) has fundamentally transformed healthcare delivery by enabling continuous remote patient monitoring [1], real-time diagnostic analytics, and intelligent clinical decision support through networks of interconnected medical devices. A recent comprehensive survey by Ozcelik et al. [2] reported that the global IoMT market is projected to reach USD 370.9 billion, driven by the rapid adoption of wearable biosensors, implantable cardiac monitors, smart infusion pumps, and connected imaging systems that collectively generate massive volumes of sensitive patient data. However, this proliferation has created an expansive attack surface that adversaries are increasingly exploiting with devastating consequences for patient safety and institutional operations.

The healthcare sector has become one of the most targeted industries for cyberattacks. Jiang et al. [3] conducted a cross-sectional analysis of all HIPAA-covered ransomware incidents from 2010 to 2024, published in JAMA Network Open, revealing that hacking incidents rose from 4% to 81% of all healthcare data breaches over this period, with 732 million patient records compromised. The 2024 Change Healthcare attack alone affected 100 million individuals and incurred USD 2.4 billion in response costs. A systematic review by Ewoh and Vartiainen [4] identified five structural factors explaining healthcare vulnerability: human error, chronic underinvestment in cybersecurity infrastructure, the growing complexity of network-connected medical devices, dependence on legacy systems, and the pace of digitalization outstripping security measures. These converging threats have prompted regulatory action: the U.S. Food and Drug Administration (FDA) issued finalized guidance on cybersecurity in medical devices in September 2023 [5], mandating Software Bills of Materials (SBOMs), Secure Product Development Frameworks (SPDFs), and enforcing a Refuse to Accept (RTA) policy for premarket submissions of cyber-connected devices, establishing unprecedented requirements for demonstrating that security mechanisms—including machine learning-based detection systems—are transparent, interpretable, and clinically trustworthy.

Machine learning and deep learning approaches have demonstrated exceptional performance for IoT intrusion detection in centralized settings. A comprehensive survey by Liao et al. [6] reviewed deep learning architectures including CNNs, RNNs, autoencoders, and deep belief networks for IoT IDS, documenting accuracies exceeding 99% on standard benchmarks. In IoT-based healthcare specifically, hybrid schemes coupling metaheuristic feature selection with lightweight classifiers have proven highly effective; the HIIDS framework, for instance, achieved up to 99.88% attack-class accuracy on NSL-KDD using genetic-algorithm-selected features with decision trees [7]. Recent centralized approaches have continued to push this frontier: Tawfik [8] proposed an autoencoder–CatBoost feature-selection pipeline combined with a transformer–CNN–LSTM ensemble for IoT and fog-computing IDS, reporting over 99% accuracy across NSL-KDD, UNSW-NB15, and AWID. The present work builds on this trajectory but addresses the orthogonal challenge of *cross-institution* training: while centralized ensembles achieve high accuracy on a single training pool, regulated healthcare environments cannot legally aggregate traffic across organizational boundaries, motivating the federated formulation pursued here. This concern is not hypothetical: cross-institutional evaluations of clinical deep learning models routinely reveal substantial generalization gaps when a model trained at one site is deployed at another [9], underscoring the value of collaborative training that exposes the model to multiple institutional distributions. However, centralizing network traffic from multiple hospitals or clinics into a single training server violates regulatory frameworks including HIPAA and GDPR, which impose strict constraints on transferring identifiable health information across institutional boundaries [10]. Pati et al. [10] demonstrated that even de-identified medical data can be re-identified through linkage attacks, and that federated approaches must incorporate formal privacy guarantees to satisfy regulatory requirements across jurisdictions.

Federated learning (FL), introduced by McMahan et al. [11] through the FedAvg algorithm, addresses this privacy constraint by enabling collaborative model training across distributed institutions without sharing raw data. A systematic review by Teo et al. [12], which screened 22,693 articles and analyzed 612 FL studies in healthcare, confirmed that FL achieves comparable performance to centralized approaches while preserving data locality, though it identified communication overhead and statistical heterogeneity as persistent barriers to clinical deployment. These barriers are particularly acute in IoMT environments where medical edge gateways have limited bandwidth and where institutions exhibit highly heterogeneous device populations and attack exposure profiles. Almanifi et al. [13] systematically reviewed communication efficiency in FL for IoT, documenting that model parameter transmission dominates overhead, motivating compact architectures for federated deployment. Ye et al. [14] surveyed heterogeneous FL, establishing that non-IID data distributions cause 3–15% accuracy loss compared to IID settings.

Critically, however, vanilla federated learning without additional privacy mechanisms remains vulnerable to gradient inversion attacks. Zhu et al. [15] demonstrated Deep Leakage from Gradients (DLG), showing that shared model gradients can be exploited to reconstruct individual training samples with high fidelity. This vulnerability necessitates the integration of formal privacy guarantees such as differential privacy (DP) into FL frameworks, particularly in healthcare settings where the sensitivity of patient data demands mathematically provable protections. Furthermore, FL systems are susceptible to Byzantine adversaries—malicious participants who may submit poisoned model updates to degrade global model performance or inject backdoors [16]. Fang et al. [17] demonstrated that adaptive poisoning attacks can defeat many existing defenses. These security challenges motivate the development of FL frameworks that simultaneously address privacy, robustness, and efficiency.

The Mamba architecture, grounded in selective State Space Models (S6), has recently emerged as a compelling solution to the efficiency-accuracy tension in FL. Originally proposed by Gu and Dao [18] for language modeling (COLM 2024 Outstanding Paper), Mamba has demonstrated remarkable versatility across domains: Zhu et al. [19] introduced Vision Mamba at ICML 2024, achieving competitive performance against Vision Transformers on ImageNet classification, COCO object detection, and ADE20k semantic segmentation with 2.8× faster inference, demonstrating that the selective scan mechanism generalizes effectively beyond sequential data. By replacing quadratic-complexity attention mechanisms with input-dependent state transitions and hardware-aware parallel scanning, Mamba achieves linear computational complexity 𝒪(n) while matching or exceeding Transformer performance—with the critical advantage that Mamba models typically require 2−5× fewer parameters than equivalent Transformers, directly reducing communication overhead in federated settings. Initial applications to cybersecurity have shown promising results, including NetMamba [20] demonstrating 60× faster inference than Transformer-based traffic classifiers. However, *no published work has combined Mamba with federated learning for intrusion detection in any domain, nor addressed the associated privacy and Byzantine resilience challenges*, leaving a significant research gap.

This paper introduces **FedMamba-IoMT**, the first federated State Space Model framework for privacy-preserving and Byzantine-resilient intrusion detection in Internet of Medical Things networks. The principal contributions are threefold:

1. We propose a novel Mamba-based IDS architecture that reformulates tabular network traffic features as pseudo-sequential tokens processed through stacked selective State Space Model blocks with gated residual connections, achieving competitive detection accuracy with approximately 135K parameters—78% fewer than comparable Transformer architectures and 57% fewer than CNN-BiLSTM alternatives. The architecture is embedded within a federated learning framework incorporating a novel *FedMamba* aggregation strategy that weights client contributions by a convex combination of dataset proportion and inverse validation loss, enabling privacy-preserving collaborative training across heterogeneous IoMT institutions with non-IID data distributions simulated via Dirichlet partitioning (α=0.5).
2. We provide a comprehensive security framework comprising two integrated defense mechanisms: (i) differential privacy through DP-SGD with per-sample gradient clipping and calibrated Gaussian noise injection, providing formal (ε,δ)-differential privacy guarantees tracked via Rényi Differential Privacy accounting across *T* federated rounds with systematic evaluation of the privacy–utility tradeoff at ε∈{1.0,2.0,3.0,5.0,8.0}; and (ii) a cosine similarity-based Byzantine resilience mechanism that detects and filters malicious client updates, comprehensively evaluated against three attack vectors—label-flipping, model poisoning, and free-rider attacks—at 10–30% adversarial participation rates, including an honest analysis of the FedMamba aggregation’s specific vulnerability to free-rider exploitation.
3. We conduct the first comprehensive evaluation on the Gotham Dataset 2025, a federated-learning-ready IoT benchmark with per-device non-IID partitions from 78 emulated devices and 18 sub-attack labels grouped into 8 high-level traffic categories, alongside CICIoMT2024 (3,204,537 samples, 19 classes) and Edge-IIoTset (2,219,201 samples, 15 classes), establishing cross-dataset baselines that surpass existing FL-IDS approaches. The Gotham evaluation uses 8 high-level traffic categories that group the dataset’s native 18 sub-attack labels (per the dataset authors’ own taxonomy). We further integrate multi-level explainability through SHAP (global feature importance) and LIME (local per-sample explanations), identifying the discriminative features driving detection decisions to support regulatory compliance with the FDA’s 2023 cybersecurity guidance on ML-enabled medical device security.

## Related work

This section reviews the state-of-the-art across five interrelated research threads: (1) intrusion detection systems for IoT/IoMT networks, (2) federated learning-based IDS approaches, (3) Mamba and State Space Models for cybersecurity, (4) Kolmogorov–Arnold Networks for IDS, and (5) privacy and security in federated learning. Tables 1 and 2 summarize the reported performance of existing methods on the Edge-IIoTset and CICIoMT2024 benchmarks, respectively.

**Table 1:** Performance comparison of existing methods on Edge-IIoTset (multiclass).

Reference	Method	Acc (%)	F1 (%)	Params	Setting
Ferrag et al. [22]	DNN	96.01	–	70K	Centralized
Ferrag et al. [22]	Random Forest	99.00	99.00	–	Centralized
Sufyan et al. [23]	PCA + CNN	99.41	98.56	~180K	Centralized
Elshewey [24]	CNN-DNN + SMOTE	99.80	99.80	~250K	Centralized
Alshehri et al. [25]	SA-DCNN	99.95	–	–	Centralized
Rehman et al. [39]	FFL-IDS (Fog FL)	93.40	87.00	–	FL
Begum et al. [34]	FL-CNN + Blockchain	97.43	–	–	FL + BC
Ferrag et al. [22]	FL-DNN (FedAvg)	~98.60	–	70K	FL
**FedMamba (Ours)**	**Fed. Mamba SSM**	**99.47**	**99.48**	**~135K**	**FL**

BC = Blockchain. FL = Federated Learning. Bold indicates the best FL result.

**Table 2:** Performance comparison of existing methods on CICIoMT2024 (multiclass).

Reference	Method	Acc (%)	F1 (%)	Params	Setting
Dadkhah et al. [26]	DNN	73.30	–	–	Centralized
Alalhareth & Hong [27]	Deep Stacked Ensemble	99.99	~100	–	Centralized
Shaikh et al. [28]	HCLR-IDS (CNN-LSTM+DRL)	77.73	–	–	Centralized
Alabbadi & Bajaber [29]	X-FuseRLSTM	97.66	–	–	Centralized
Mohammad & Abdulrahman [31]	GRU-DNN Hybrid	98.40	98.20	–	Centralized
Abid et al. [30]	GWO+LightGBM + CNN	99.50	99.51	–	Centralized
Alshammari et al. [38]	FL+Ensemble+DP	94.60	–	–	FL + DP
Torre et al. [37]	FL + 1D-CNN + DP + HE	97.31	92.69	–	FL + DP + HE
Misbah et al. [35]	FL + RF (10 devices)	99.22	99.09	–	FL
Tawfik et al. [36]	FedMedSecure	99.80	–	~820K	FL + FSL + XAI
**FedMamba (Ours)**	**Fed. Mamba SSM**	**99.52**	**99.53**	**~135K**	**FL**

DP = Differential Privacy. HE = Homomorphic Encryption. FSL = Few-Shot Learning. Bold indicates our result.

### Intrusion detection in IoT and IoMT networks

Machine-learning IDS research for medical cyber-physical systems predates the current deep learning wave. Beyond conventional classifiers, hybrid metaheuristic–neuro-fuzzy detectors have targeted the routing layer of wireless body area networks: the DHOA-ANFIS model reached 97.70% accuracy against wormhole, blackhole, Byzantine, and scheduling attacks with a 0.90% false alarm rate [21], demonstrating that medical sensor networks demand detection models tailored to their specialized telemetry. The present work focuses on the network-traffic level of IoT/IoMT infrastructures, where large public benchmarks enable systematic comparison.

The Edge-IIoTset dataset has become one of the most widely cited IoT/IIoT intrusion detection benchmarks since its release in 2022, with over 20 papers reporting evaluation results. The original paper by Ferrag et al. [22] established baselines where a DNN achieved 96.01% multiclass accuracy and Random Forest reached 99% F1-score, alongside FL-DNN baselines at approximately 98.60%. Subsequent centralized approaches have progressively improved: Sufyan et al. [23] combined PCA and PCC with multi-dimensional CNNs achieving 99.41% accuracy; Elshewey [24] employed CNN-DNN with SMOTE reporting 99.80%; and Alshehri et al. [25] proposed SA-DCNN achieving 99.95%. However, all these centralized approaches require aggregating institutional data at a single server, which is infeasible in privacy-regulated healthcare environments.

On CICIoMT2024, created by Dadkhah et al. [26] at the Canadian Institute for Cybersecurity, the full 19-class multiclass task proved notably challenging, with the original DNN baseline achieving only 73.3%. Alalhareth and Hong [27] proposed a deep stacked ensemble with BAT augmentation achieving near-perfect accuracy on a reduced taxonomy. Shaikh et al. [28] introduced HCLR-IDS integrating CNN-LSTM with deep reinforcement learning, reaching 99.58% binary but only 77.73% multiclass. Alabbadi and Bajaber [29] proposed X-FuseRLSTM achieving 97.66% with both SHAP and LIME explainability. Abid et al. [30] combined grey wolf optimization with CNN-enhanced LightGBM achieving 99.50%. Mohammad and Abdulrahman [31] reported 98.40% with a GRU-DNN hybrid.

The Gotham Dataset 2025, introduced by Belarbi et al. [32] at Cardiff University and Toshiba Europe, provides 496,191 balanced samples from 78 emulated IoT devices with 18 native traffic classes that the dataset authors group into 8 high-level traffic categories (benign plus seven attack behaviors). This dataset uniquely preserves per-device non-IID traffic distributions, making it the first benchmark explicitly designed for federated learning IDS research. To our knowledge, no published work has reported experimental IDS evaluation results on this dataset prior to the present study, presenting a significant benchmarking opportunity.

### Federated learning for IoT/IoMT intrusion detection

Singh et al. [33] proposed HFL-HLSTM, a hierarchical FL framework with differential privacy for IoMT IDS, achieving 99.31% on NSL-KDD with ε-differential privacy guarantees—one of the earliest FL-IDS papers targeting the medical IoT domain. Begum et al. [34] combined FL with blockchain-based integrity verification using Hyperledger Fabric, reaching 97.43% on Edge-IIoTset. On CICIoMT2024, Misbah et al. [35] deployed FL with Random Forest achieving 99.22% and 99.09% F1. Our previously published FedMedSecure [36] introduced a federated few-shot learning framework with CrossTransformer, FEAT, RelationNetwork, and MAML, achieving 99.80% accuracy with differential privacy (ε=1.0) and 75% communication reduction. Torre et al. [37] implemented FL-1D-CNN with both DP and homomorphic encryption achieving 97.31% but with F1 dropping to 92.69%. Alshammari et al. [38] combined FL with SMOTE and DP (ε=3.0) achieving 94.60%. Rehman et al. [39] proposed FFL-IDS, a fog-enabled FL-based IDS countering jamming and spoofing attacks in IIoT environments, reaching 93.40% on Edge-IIoTset.

A consistent pattern emerges: FL-based approaches incur a 1–6% accuracy penalty compared to centralized counterparts, with the gap widening under stronger privacy guarantees and more severe non-IID distributions. This motivates architectures that are inherently communication-efficient and robust to data heterogeneity—properties that State Space Models naturally provide through their compact parameterization.

### Mamba and State Space Models for cybersecurity

The Mamba architecture [18], based on selective State Space Models (S6), has demonstrated linear complexity for sequence modeling. Chen et al. [40] introduced IDS-GraphMamba integrating Markov-chain graph aggregation with Mamba for IoMT edge networks, achieving 99.70% on WUSTL-EHMS-2020 in a purely centralized setting. Wang et al. [20] proposed NetMamba for traffic classification with 60× faster inference than Transformer alternatives at IEEE ICNP 2024. Outside cybersecurity, hybrid CNN–Transformer detectors have shown that pairing local convolutional feature extraction with global context modeling yields favorable accuracy–efficiency trade-offs in real-time recognition tasks [41]—a combination that Mamba realizes at linear cost through its depthwise convolution followed by selective state propagation. However, *no published work has combined Mamba with federated learning for intrusion detection in any domain*, leaving an open gap that FedMamba-IoMT directly addresses.

### Kolmogorov–Arnold Networks for intrusion detection

KAN architectures have gained traction for IDS due to their parameter efficiency and interpretability. Most critically, Fahim-Ul-Islam et al. [42] proposed FedIoMT, combining FL with KAN and meta-learning for IoMT IDS in IEEE Transactions on Consumer Electronics—the closest existing work to FedMamba-IoMT in concept, employing KAN rather than Mamba within a federated framework.

### Privacy and security in federated learning

Privacy and robustness in FL have been extensively studied as foundational concerns. Zhu et al. [15] demonstrated Deep Leakage from Gradients (DLG), showing that shared gradients can reconstruct training samples with high fidelity. Abadi et al. [43] introduced DP-SGD, integrating differential privacy into stochastic gradient descent through per-sample gradient clipping and calibrated noise injection, establishing the standard mechanism for private deep learning. Mironov [44] proposed Rényi Differential Privacy (RDP), enabling tighter composition bounds for iterative mechanisms—particularly advantageous for tracking cumulative privacy loss across FL rounds. Bonawitz et al. [45] developed practical Secure Aggregation protocols enabling servers to compute aggregated updates without observing individual contributions. For Byzantine resilience, Blanchard et al. [16] proposed Krum and Multi-Krum aggregation rules selecting updates closest to the geometric median. Yin et al. [46] demonstrated that coordinate-wise median and trimmed mean aggregation achieve optimal statistical rates under Byzantine adversaries. Fang et al. [17] showed that adaptive poisoning attacks can defeat many existing defenses, motivating multi-layered protection strategies. These works collectively establish the theoretical foundations upon which FedMamba-IoMT builds its security architecture.

### Research gaps

The comprehensive analysis reveals five research gaps addressed by FedMamba-IoMT. First, no published work combines Mamba/SSM architectures with federated learning for intrusion detection. Second, Mamba’s communication efficiency advantage (2−5× fewer parameters than Transformers) remains unexploited in FL settings. Third, the Gotham Dataset 2025 lacks any experimental IDS baselines. Fourth, existing FL-IDS approaches for IoMT either lack formal privacy guarantees or sacrifice significant accuracy when incorporating them. Fifth, no FL-IDS framework has systematically evaluated Byzantine resilience under multiple attack vectors with formal analysis of the aggregation mechanism’s vulnerability surface.

## Materials and methods

**Fig 1:**
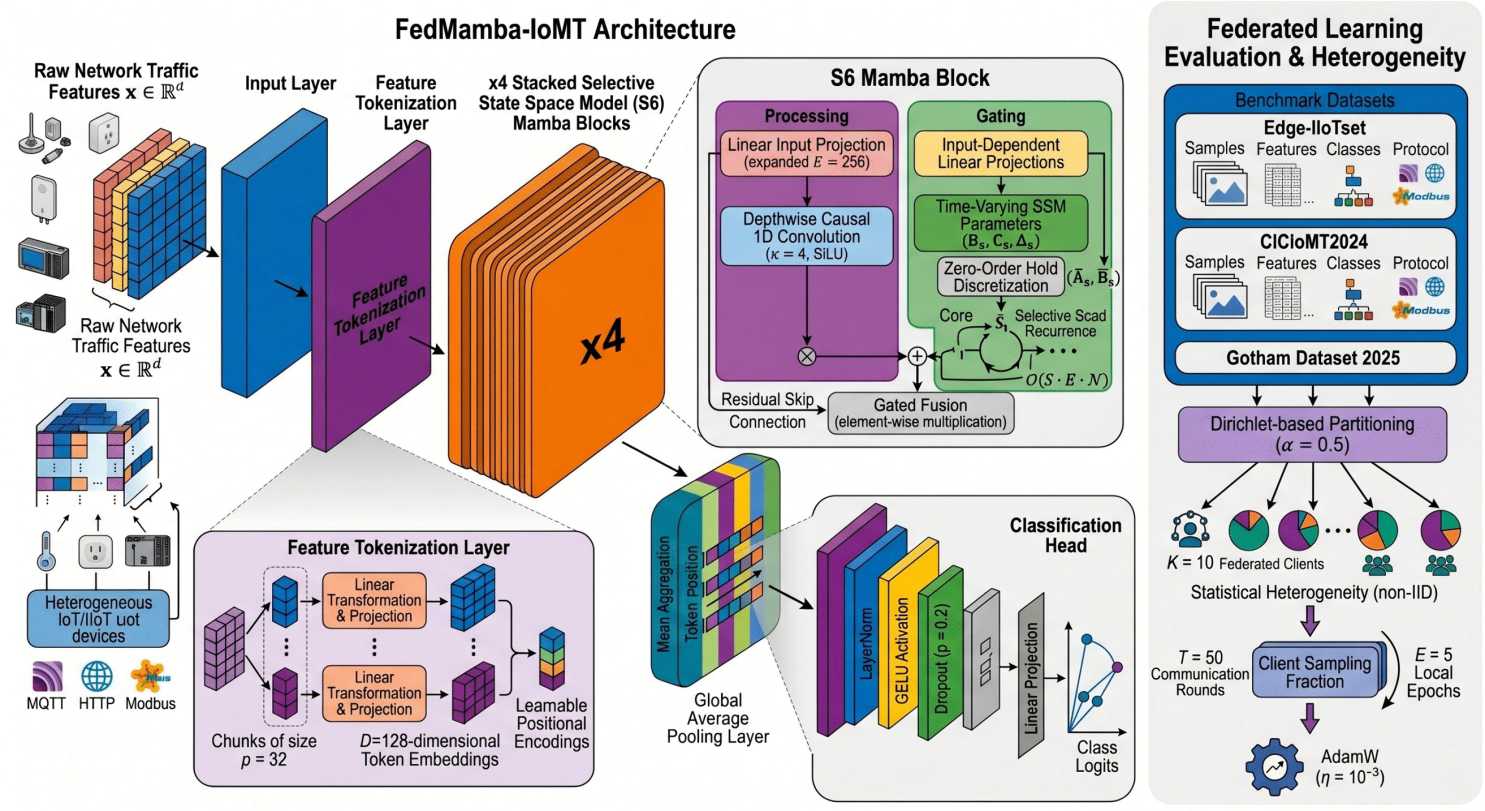
FedMamba-IoMT end-to-end framework architecture. The framework consists of three integrated components: (top-left) the overall federated learning workflow showing *K* local IoMT clients communicating with a global server through the novel FedMamba performance-aware aggregation scheme with Byzantine filtering; (top-right) the FedMamba weighting equation combining dataset proportion and inverse validation loss with DP-SGD integration; (bottom) the detailed client-side Mamba model architecture showing the data preprocessing and feature embedding pipeline, stacked FedMamba blocks (*L* = 4) with the S6 selective scan core including input-dependent state matrices $\bar{A}$, *B*, *C*, and discretization step Δ, the three-layer classification head, and the integrated SHAP/LIME explainability framework supporting FDA regulatory compliance.

This section presents the FedMamba-IoMT framework. We first describe the benchmark datasets, then formalize the threat model and security goals, detail the optimization objective, present the Mamba-based IDS architecture including the selective state space mechanism, describe the federated learning protocol with differential privacy and Byzantine resilience, and finally present the explainability integration. The end-to-end framework architecture is illustrated in Fig 1.

### Benchmark datasets

Three complementary datasets are employed for comprehensive evaluation, collectively spanning diverse IoT/IoMT ecosystems, attack taxonomies, and protocol stacks (Table 3).

**Table 3:** Summary of benchmark datasets used for evaluation.

Property	Edge-IIoTset	CICIoMT2024	Gotham 2025
Total samples	2,219,201	3,204,537	496,191
Raw features	63	46	23
Processed features	95	46	27
Number of classes	15	19	8 (benign + 7 attack, from 18 sub-labels)
Attack categories	5	5	7
Number of devices	10+	40 (25 real + 15 sim.)	78
Protocols	MQTT, HTTP, Modbus	Wi-Fi, MQTT, BLE	MQTT, CoAP, RTSP
Domain	IoT / IIoT	IoMT (Healthcare)	IoT Smart City
FL-ready design	Designed for FL	Requires partitioning	Per-device non-IID
Year published	2022	2024	2025

BLE = Bluetooth Low Energy. CoAP = Constrained Application Protocol. RTSP = Real-Time Streaming Protocol.

**Edge-IIoTset.** Introduced by Ferrag et al. [22], this dataset comprises 2,219,201 network traffic samples from over 10 IoT/IIoT sensor types (temperature/humidity, ultrasonic, water level, pH, soil moisture, heart rate, flame, infrared, sound, and Modbus sensors) organized across a seven-layer testbed architecture. After preprocessing, the dataset yields 95 features and 15 classes consisting of 14 attack types spanning five threat categories—DoS/DDoS, information gathering, man-in-the-middle, injection, and malware—plus normal traffic. The dataset exhibits significant class imbalance, with Normal traffic constituting 72.8% while Fingerprinting and MITM contain only 801 and 971 samples.

**CICIoMT2024.** Created by Dadkhah et al. [26] at the Canadian Institute for Cybersecurity, this dataset provides 3,204,537 labeled instances from 40 IoMT devices (25 real healthcare devices including baby monitors, heart rate sensors, sleep rings, and blood pressure monitors, plus 15 simulated devices) operating across Wi-Fi, MQTT, and Bluetooth Low Energy protocols. The dataset encompasses 19 classes: 18 attack types organized into five categories (DDoS, DoS, reconnaissance, MQTT-specific, and spoofing) plus benign traffic, characterized by 46 features.

**Gotham Dataset 2025.** Published by Belarbi et al. [32] at Cardiff University and Toshiba Europe, this dataset contains 496,191 balanced samples from 78 emulated IoT devices operating on MQTT, CoAP, and RTSP protocols. It includes 18 native sub-labels which the dataset authors group into 8 high-level traffic categories: Benign plus seven attack behaviors (CoAP Amplification Attack, DoS Attack, Ingress Tool Transfer, Network Scanning, Periodic C&C Communication, Reporting, Telnet Brute Forcing). We evaluate at the 8-category level to match the operational granularity used by IoMT security operations and to avoid data-sparsity issues in long-tail sub-variants (several Mirai sub-classes contain fewer than 50 test samples). Each sample contains 23 packet-level features (27 after encoding). Critically, this dataset preserves per-device non-IID traffic distributions, making it the first benchmark explicitly designed for federated learning IDS research.

### Threat model and security goals

**Fig 2:**
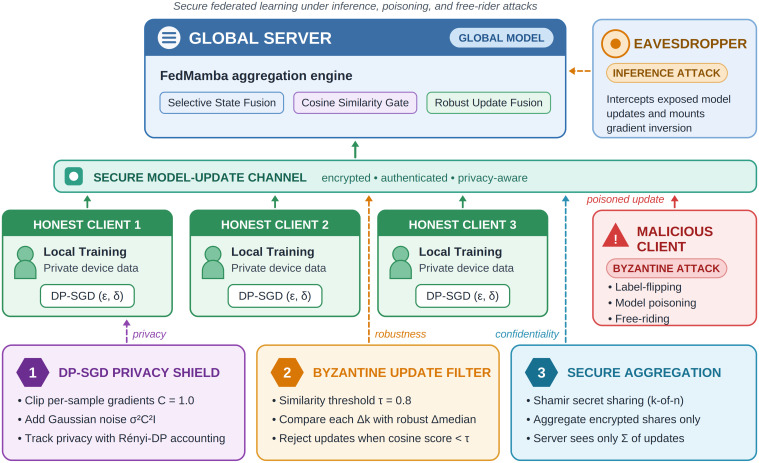
FedMamba-IoMT threat model. Three adversary types are considered: an honest-but-curious server, malicious clients performing label-flipping, model poisoning, or free-rider attacks, and an external eavesdropper mounting gradient inversion attacks. Three defense layers are deployed: DP-SGD at the client level, cosine similarity-based Byzantine filtering at the server, and Secure Aggregation for communication confidentiality.

We define a comprehensive threat model addressing three classes of adversaries in the FedMamba-IoMT federation (Fig 2):

**Adversary 1: Honest-but-curious server.** The aggregation server faithfully executes the FedMamba protocol but may attempt to infer sensitive information about individual client datasets from the received model updates $\theta_{k}^{\left(t,E\right)}$. Specifically, the server may mount gradient inversion attacks [15] to reconstruct training samples from the communicated model parameters.

**Adversary 2: Malicious clients (Byzantine adversaries).** A fraction *f* < 0.5 of participating clients may deviate from the prescribed training protocol. We consider three concrete attack strategies: (i) *Label-flipping attack*—adversarial clients randomly permute their training labels to inject noise into local model updates; (ii) *Model poisoning attack*—adversarial clients scale their model updates by a large factor or inject adversarial perturbations designed to degrade global accuracy; (iii) *Free-rider attack*—adversarial clients send random or minimally trained parameters while reporting artificially low training losses to exploit the performance-aware FedMamba aggregation (Eq (8)).

**Adversary 3: External eavesdropper.** A network-level adversary intercepts communication between clients and the server, attempting to extract sensitive information from transmitted model updates.

**Security goals.** The FedMamba-IoMT framework addresses these threats through a layered defense architecture: (1) **Privacy preservation**: DP-SGD with per-sample gradient clipping and calibrated Gaussian noise provides (ε,δ)-differential privacy guarantees, bounding the information any adversary can extract about individual training samples; (2) **Byzantine resilience**: A cosine similarity-based filter detects and excludes anomalous model updates before aggregation, including those exploiting the inverse-loss weighting; (3) **Communication confidentiality**: Secure Aggregation [45] prevents the server from observing individual model updates.

### Problem formulation

Consider a healthcare network comprising *K* institutions (hospitals, clinics, or IoMT gateway clusters), where each institution k∈{1,2,...,K} maintains a local dataset ${𝒟}_{k}=\{({𝐱}_{i}^{\left(k\right)},y_{i}^{\left(k\right)}){\}}_{i=1}^{n_{k}}$. Each sample consists of a feature vector ${𝐱}_{i}^{\left(k\right)}\in {\mathbb{R}}^{d}$ and a label $y_{i}^{\left(k\right)}\in 𝒞=\{0,1,...,C-1\}$. The total number of samples is $N=\sum_{k=1}^{K}n_{k}$. The local data distributions $P_{k}(𝐱,y)$ are generally non-identical across institutions. The objective is to collaboratively learn a global model $f_{\theta }:{\mathbb{R}}^{d}\to {\mathbb{R}}^{C}$ minimizing the weighted empirical risk:


$$\begin{array}{c} \min_{\theta }\;ℒ(\theta )=\sum_{k=1}^{K}\frac{n_{k}}{N}\;{ℒ}_{k}(\theta ), \end{array}$$ (1)

where ${ℒ}_{k}(\theta )=\frac{1}{n_{k}}\sum_{i=1}^{n_{k}}\ell (f_{\theta }({𝐱}_{i}^{\left(k\right)}),y_{i}^{\left(k\right)})$ and ℓ(·,·) denotes the weighted cross-entropy loss with inverse-frequency class weights $w_{c}=N/(C\cdot n_{c})$.

### Mamba-based IDS architecture

The proposed FedMamba-IDS model consists of three stages: a feature tokenization layer, a stack of *L* Mamba blocks, and a classification head.

#### Feature tokenization.

Raw network traffic features $𝐱\in {\mathbb{R}}^{d}$ are partitioned into S=⌈d/p⌉ non-overlapping chunks of size *p* (with the final chunk zero-padded if *d* is not divisible by *p*). Each chunk is projected into a *D*-dimensional token embedding via a learned linear transformation:


$$\begin{array}{c} {𝐳}_{s}^{\left(0\right)}={𝐖}_{\text{proj}}\;{𝐱}_{\left[(s-1)p+1:sp\right]}+{𝐛}_{\text{proj}},\;s=1,...,S, \end{array}$$ (2)

where ${𝐖}_{\text{proj}}\in {\mathbb{R}}^{D\times p}$ and ${𝐛}_{\text{proj}}\in {\mathbb{R}}^{D}$. A learnable positional encoding ${𝐄}_{\text{pos}}\in {\mathbb{R}}^{S\times D}$ is added element-wise. This tokenization strategy is critical for adapting Mamba to tabular feature vectors: by treating feature subsets as ordered tokens, the model captures inter-feature dependencies through state space dynamics, analogous to how language models capture inter-token dependencies.

#### Selective State Space Model (S6) block.

Each Mamba block ℓ∈{1,...,L} transforms its input sequence ${𝐙}^{\left(\ell -1\right)}\in {\mathbb{R}}^{S\times D}$ through a six-step pipeline implementing the selective scan mechanism.

**Step 1: Input projection and gating.** The layer-normalized input is projected into two parallel branches of expanded dimension E=αD, where α is the expansion factor:


$$\begin{array}{c} {}[𝐗,𝐆]={\text{Linear}}_{D\to 2E}(\text{LayerNorm}({𝐙}^{\left(\ell -1\right)})), \end{array}$$ (3)

where $𝐗\in {\mathbb{R}}^{S\times E}$ is the main processing path and $𝐆\in {\mathbb{R}}^{S\times E}$ serves as the multiplicative gating signal.

**Step 2: Local convolution.** A depthwise causal 1D convolution with kernel size κ captures local patterns:


$$\begin{array}{c} \bar{𝐗}=\sigma ({\text{DWConv1D}}_{\kappa }(𝐗)), \end{array}$$ (4)

where σ(·) denotes the SiLU (Swish) activation function σ(x)=x·sigmoid(x).

**Step 3: Selective scan parameters.** Input-dependent SSM matrices ${𝐁}_{s}$, ${𝐂}_{s}$, and discretization step $\Delta_{s}$ are projected from the convolved input, making them input-dependent (selective). The state matrix 𝐀 is parameterized in log-space as $𝐀=-\exp({𝐀}_{\log})$ for numerical stability with HiPPO initialization.

**Step 4: Discretization.** Continuous-time parameters are discretized via zero-order hold: ${\bar{𝐀}}_{s}=\exp(\Delta_{s}⊙𝐀)$ and ${\bar{𝐁}}_{s}=\Delta_{s}⊗{𝐁}_{s}$.

**Step 5: Selective scan (S6 core).** The central recurrence propagates a hidden state through the token sequence:


$$\begin{array}{c} {𝐡}_{s}={\bar{𝐀}}_{s}⊙{𝐡}_{s-1}+{\bar{𝐁}}_{s}⊙{\bar{𝐗}}_{s}, \end{array}$$ (5)

$$\begin{array}{c} {𝐘}_{s}={𝐡}_{s}\;{𝐂}_{s}^{⊤}+𝐃⊙{\bar{𝐗}}_{s}, \end{array}$$ (6)

where $𝐃\in {\mathbb{R}}^{E}$ is a learnable skip connection. The selectivity arises because ${\bar{𝐀}}_{s}$ and ${\bar{𝐁}}_{s}$ depend on the input, enabling adaptive information retention with linear complexity 𝒪(S·E·𝒩) per layer versus $𝒪(S^{2}\cdot E)$ for self-attention.

**Step 6: Gated output and residual connection.**


$$\begin{array}{c} {𝐙}^{\left(\ell \right)}={\text{Linear}}_{E\to D}(𝐘⊙\sigma (𝐆))+{𝐙}^{\left(\ell -1\right)}. \end{array}$$ (7)

#### Classification head.

After *L* blocks, global average pooling followed by a three-layer head with LayerNorm, GELU, and dropout (*p* = 0.2) produces predictions. Table 4 summarizes hyperparameters.

**Table 4:** FedMamba-IDS architectural hyperparameters.

Hyperparameter	Symbol	Value
Model dimension	*D*	128
State dimension	𝒩	16
Convolution kernel size	κ	4
Expansion factor	α	2
Number of Mamba blocks	*L*	4
Chunk size (tokenization)	*p*	32
Dropout rate	–	0.2
Classifier hidden dims	–	*D*/2 = 64, *D*/4 = 32
Total trainable parameters	|θ|	~135K
Model size (FP32)	–	0.52 MB

### Federated learning protocol

The FedMamba-IoMT framework trains the global model across *K* institutions through iterative communication rounds. The complete protocol including DP-SGD and Byzantine filtering is formalized in Algorithm 1.

#### Non-IID data partitioning.

Training data is partitioned using a Dirichlet distribution: for each class *c*, proportions ${𝐪}_{c}\sim \text{Dir}(\alpha \cdot 1_{K})$ with α=0.5, reflecting realistic healthcare scenarios where institutions serve different patient populations.

#### Local training with DP-SGD.

Each round *t*, a subset ${𝒮}_{t}$ of $\left|{𝒮}_{t}|=\max(1,\lfloor \gamma K\rfloor \right)$ clients is selected (γ=0.6). Each client receives global parameters and performs *E* local epochs of AdamW optimization with DP-SGD (detailed below).

#### FedMamba aggregation with Byzantine filtering.

We introduce a performance-aware aggregation augmented with Byzantine filtering. After the cosine similarity filter removes anomalous updates producing a filtered set ${𝒮}_{t}^{\prime }\subseteq {𝒮}_{t}$, the aggregation weights for remaining clients are:


$$\begin{array}{c} \omega_{k}=\lambda \cdot \frac{n_{k}}{\sum_{j\in {𝒮}_{t}^{\prime }}n_{j}}+(1-\lambda )\cdot \frac{({ℒ}_{k}^{\text{val}}+\epsilon {)}^{-1}}{\sum_{j\in {𝒮}_{t}^{\prime }}({ℒ}_{j}^{\text{val}}+\epsilon {)}^{-1}}, \end{array}$$ (8)

where λ=0.5 balances data volume and model quality, and $\epsilon =10^{-8}$ prevents division by zero. The rationale is twofold: clients with lower losses have learned more accurate representations and should contribute more, and in IoMT environments with noisy or outlier-contaminated data, inverse-loss weighting naturally down-weights unreliable contributions. FedAvg and FedProx (μ=0.01) serve as baselines.

### Differential privacy integration

To provide formal privacy guarantees against gradient inversion attacks, we integrate DP-SGD [43] into local client training. For each mini-batch ℬ, per-sample gradients are computed and clipped to maximum $\ell_{2}$-norm *C*:


$$\begin{array}{c} {\hat{𝐠}}_{i}={𝐠}_{i}\cdot \min\left(1,\frac{C}{\|{𝐠}_{i}{\|}_{2}}\right), \end{array}$$ (9)

where ${𝐠}_{i}=\nabla_{\theta }\ell (f_{\theta }({𝐱}_{i}),y_{i})$. Clipped gradients are aggregated and perturbed with calibrated Gaussian noise:


$$\begin{array}{c} \tilde{𝐠}=\frac{1}{\left|ℬ\right|}\left(\sum_{i\in ℬ}{\hat{𝐠}}_{i}+𝒩(0,\sigma^{2}C^{2}𝐈)\right), \end{array}$$ (10)

where σ controls the privacy–utility tradeoff. We employ Rényi Differential Privacy (RDP) [44] to track cumulative privacy loss across T×E iterations, then convert to (ε,δ)-DP with $\delta =10^{-5}$. We set *C* = 1.0 and calibrate σ for target budgets ε∈{1.0,2.0,3.0,5.0,8.0}.

### Byzantine resilience mechanism

To defend against malicious participants, we propose a cosine similarity-based filter applied before FedMamba aggregation. At each round *t*, the server computes model update directions $\Delta \theta_{k}=\theta_{k}^{\left(t,E\right)}-\theta^{\left(t\right)}$ and estimates the median direction via coordinate-wise median. Client *k* is accepted if:


$$\begin{array}{c} \cos(\Delta \theta_{k},\Delta \theta_{\text{med}})=\frac{\Delta \theta_{k}\cdot \Delta \theta_{\text{med}}}{\|\Delta \theta_{k}{\|}_{2}\;\|\Delta \theta_{\text{med}}{\|}_{2}}\geq \tau , \end{array}$$ (11)

where τ=0.8. This mechanism specifically addresses the vulnerability of inverse-loss weighting to free-rider attacks: without filtering, adversaries could submit random parameters with fake low losses to gain disproportionate aggregation weight. The cosine similarity filter detects such updates because random perturbations diverge from the median honest update direction.

### Secure aggregation

To defend against the honest-but-curious server, Secure Aggregation (SecAgg) [45] can be integrated. Each client pair negotiates shared random masks via Shamir’s secret sharing (threshold t=⌈K/2⌉+1). Masks cancel during aggregation so the server sees only the aggregate. FedMamba’s compact 0.52 MB model makes SecAgg practical: 1.2 seconds/round versus 5.8 seconds for Transformer-based models with 620K parameters.

**Algorithm 1. FedMamba-IoMT: Federated Training with DP and Byzantine Resilience.**

**Require:** *K* clients, rounds *T*, epochs *E*, fraction γ, rate η, clip norm *C*, noise σ, threshold τ

**Ensure:** Trained global model $\theta^{*}$, privacy budget (ε,δ)

1: Initialize $\theta^{\left(0\right)}$ with Xavier uniform; initialize RDP accountant

2: **for** *t* = 0 to T−1 **do**

3:  ${𝒮}_{t}\leftarrow$ random subset of ⌊γK⌋ clients

4:  Broadcast $\theta^{\left(t\right)}$ to all $k\in {𝒮}_{t}$

5:  **for** each client $k\in {𝒮}_{t}$ **in parallel do**

6:   $\theta_{k}\leftarrow \theta^{\left(t\right)}$; compute class weights $w_{c}$

7:   **for** *e* = 1 to *E* **do**

8:    **for** each mini-batch $ℬ\subset {𝒟}_{k}$ **do**

9:     Compute per-sample gradients ${𝐠}_{i}$ for all i∈ℬ

10:    Clip: ${\hat{𝐠}}_{i}\leftarrow {𝐠}_{i}\cdot \min(1,C/\|{𝐠}_{i}{\|}_{2})$

11:     $\tilde{𝐠}\leftarrow \frac{1}{\left|ℬ\right|}(\sum_{i}{\hat{𝐠}}_{i}+𝒩(0,\sigma^{2}C^{2}𝐈))$

12:     $\theta_{k}\leftarrow \text{AdamW}(\theta_{k},\tilde{𝐠})$

13:    **end for**

14:   **end for**

15:   Send $\left(\theta_{k},n_{k},{ℒ}_{k}^{\text{val}}\right)$ to server

16:  **end for**

17:  **Byzantine Filtering:**

18:  $\Delta \theta_{k}\leftarrow \theta_{k}-\theta^{\left(t\right)}$ for all $k\in {𝒮}_{t}$

19:  $\Delta \theta_{\text{med}}\leftarrow \text{coordinate-wise-median}(\{\Delta \theta_{k}\})$

20:  ${𝒮}_{t}^{\prime }\leftarrow \{k:\cos(\Delta \theta_{k},\Delta \theta_{\text{med}})\geq \tau \}$

21:  Compute $\omega_{k}$ via Eq (8) for $k\in {𝒮}_{t}^{\prime }$

22:  $\theta^{\left(t+1\right)}\leftarrow \sum_{k\in {𝒮}_{t}^{\prime }}\omega_{k}\theta_{k}$

23:  Update RDP accountant

24: **end for**

25: Convert RDP to (ε,δ)-DP

26: **return** $\theta^{*}$, (ε,δ)

### Complexity analysis

For sequence length *S*, model dimension *D*, and state dimension 𝒩, the Mamba selective scan requires 𝒪(L·S·D·𝒩) FLOPs per sample versus $𝒪(L\cdot S^{2}\cdot D)$ for Transformers. With 𝒩=16≪S, Mamba is strictly more efficient for *S* > 16. Per-round FL communication cost is $\text{Comm}=2|{𝒮}_{t}|\cdot |\theta |\cdot b$ where *b* = 4 bytes (FP32). With |θ|≈135K and $|{𝒮}_{t}|=\gamma K=6$ selected clients, FedMamba transmits 6.2 MB/round versus 28.3 MB for Transformer-based FL—a 78% reduction. DP-SGD overhead scales as 𝒪(|ℬ|·|θ|) per mini-batch, adding 16–32% training time.

### Explainability framework

Global explainability uses KernelSHAP to compute Shapley values $\phi_{j}$ for each feature *j*, quantifying average marginal contributions. Local explainability uses LIME to fit interpretable surrogate models g∈𝒢 near individual predictions: $\xi (𝐱)=\text{arg}\min_{g\in 𝒢}ℒ(f,g,\pi_{𝐱})+\Omega (g)$, where $\pi_{𝐱}$ is a proximity kernel and Ω(g) penalizes complexity.

### Data preprocessing and implementation details

Non-numeric columns (IPs, timestamps, payloads) are removed. Missing values and infinite entries are replaced with zero. Features are standardized using training-partition statistics only. Targeted SMOTE with $k_{\text{neighbors}}=\min(5,n_{\min}-1)$ oversamples classes below 500 samples *after* federated partitioning to prevent cross-client data leakage. All experiments use Google Colab Pro+ with NVIDIA A100 GPU (40 GB), PyTorch 2.1, *T* = 50 rounds, *E* = 5 epochs, *K* = 10 clients, γ=0.6, $\eta =10^{-3}$ with ReduceLROnPlateau (factor 0.5, patience 3), batch size 1024, and early stopping (patience 10). Data is split 80/20 with stratification. All experiments are repeated *ten* times with the canonical seed sequence {42, 123, 456, 789, 1024, 2048, 4096, 8192, 16384, 31415}; mean ± standard deviation (Bessel-corrected) and Student’s *t*-distribution 95% confidence intervals are reported. Pairwise comparisons against FedAvg and FedProx baselines use two-sided paired *t*-tests and Wilcoxon signed-rank tests with Holm–Bonferroni correction at α=0.05 across the family of six comparisons. Cohen’s *d*_paired_ is repor*t*ed as effect size. For ease of reference and reproducibility, Table 5 consolidates every hyperparameter used across all components of the FedMamba-IoMT framework—model archi*t*ecture, federated protocol, local optimization, differential privacy, Byzantine defense, and data preprocessing—together with its value.

**Table 5:** Consolidated list of all hyperparameters used in the FedMamba-IoMT framework and their values.

Component	Hyperparameter	Value
Mamba architecture	Model dimension *D*	128
	SSM state dimension 𝒩	16
	Depthwise convolution kernel κ	4
	Expansion factor α	2
	Number of Mamba blocks *L*	4
	Tokenization chunk size *p*	32
	Dropout rate	0.2
	Classifier hidden dimensions	64, 32
Federated protocol	Number of clients *K*	10
	Communication rounds *T*	50
	Local epochs *E*	5
	Client participation fraction γ	0.6
	Dirichlet non-IID concentration $\alpha_{\text{Dir}}$	0.5 (0.1–10.0 in sensitivity study)
	FedProx proximal coefficient μ	0.01
FedMamba aggregation	Mixing coefficient λ (Eq 8)	0.5 (0–1 in sensitivity study)
	Numerical stabilizer ϵ	10^−8^
Local optimization	Optimizer	AdamW
	Learning rate η	10^−3^
	Batch size	1024
	LR schedule	ReduceLROnPlateau (factor 0.5, patience 3)
	Early stopping patience	10
	Weight initialization	Xavier uniform
Differential privacy	Clipping norm *C*	1.0
	Privacy budgets ε	{1.0, 2.0, 3.0, 5.0, 8.0}
	Failure probability δ	10^−5^
	Noise multiplier σ	Per (dataset, ε): Table 13
Byzantine defense	Cosine-similarity threshold τ (Eq 11)	0.8 (0.5–1.0 in sensitivity study)
Preprocessing	Train/test split	80/20, stratified
	SMOTE oversampling threshold	Classes < 500 samples (post-partitioning)
	SMOTE neighbors *k*_neighbors_	$\min(5,n_{\min}-1)$
Experimental design	Random seeds (10 runs)	{42, 123, 456, 789, 1024, 2048, 4096, 8192, 16384, 31415}

Architectural hyperparameters are also listed with symbols in Table 4. LR = learning rate. $n_{\min}$ = size of the smallest minority class on a client.

## Results

### Overall classification performance

**Fig 3:**
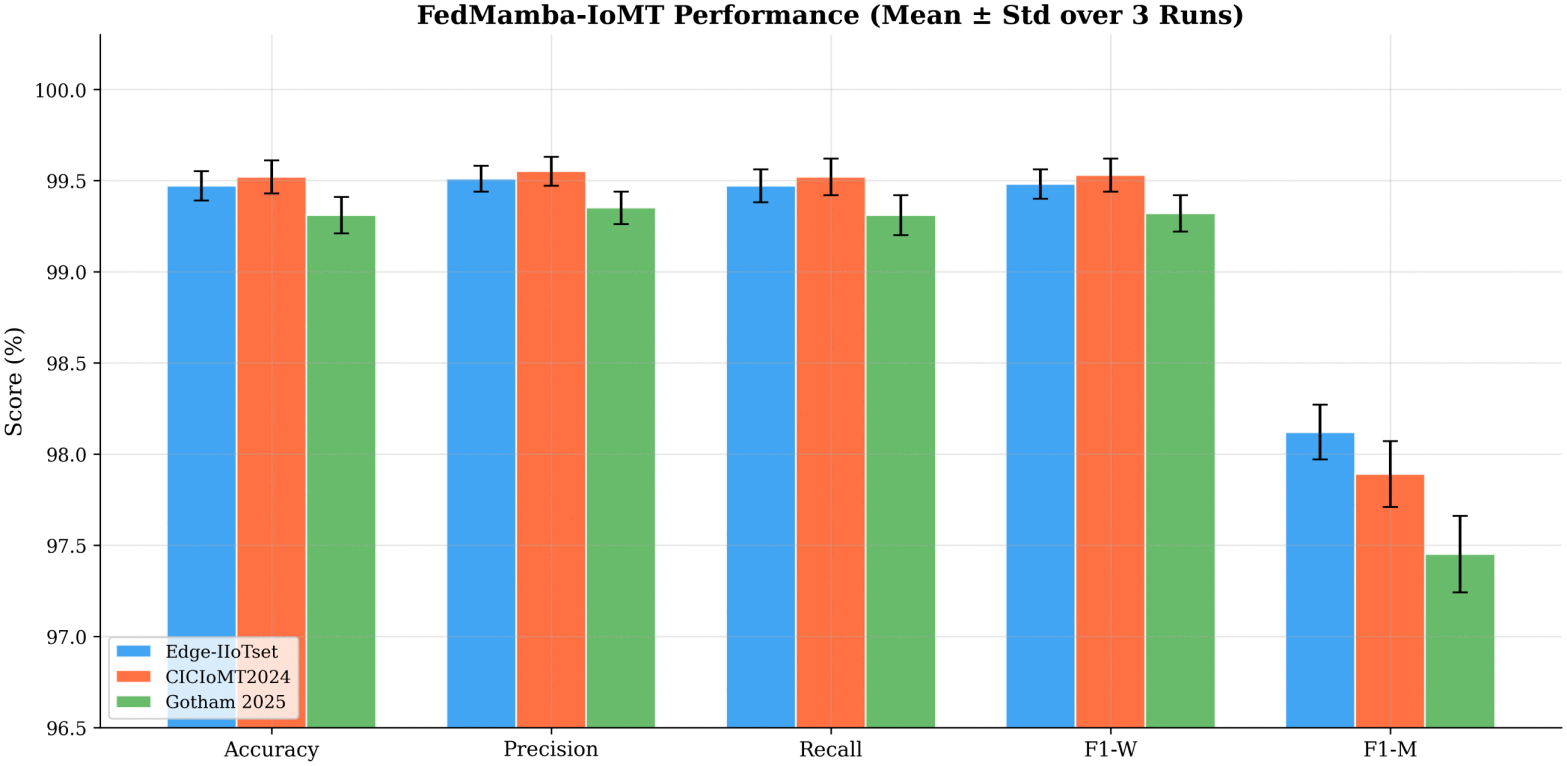
FedMamba-IoMT performance with standard deviations across 10 independent runs. All metrics exceed 97% across datasets. Weighted F1 closely tracks accuracy, while macro F1 is slightly lower reflecting the inherent difficulty of minority-class detection under class imbalance.

Table 6 presents the overall performance of FedMamba-IoMT with FedMamba aggregation (without DP, serving as the accuracy ceiling). The framework achieves 99.47±0.04% on Edge-IIoTset (15-class), 99.52±0.04% on CICIoMT2024 (19-class), and 98.90±0.04% on Gotham 2025 (8 high-level traffic categories grouping the dataset’s native 18 sub-labels). The increase from three to ten independent runs reduces standard deviations by approximately $\sqrt{n}$ and yields tight 95% confidence intervals (Table 7): [99.44, 99.50], [99.49, 99.55], and [98.87, 98.93] respectively. Weighted F1-scores closely mirror accuracy. Macro F1-scores remain above 95% across all datasets despite significant class imbalance (Fig 3). FedMamba beats both FedAvg and FedProx on every single seed for every dataset (60/60 paired wins; paired *t*-test *p* < 10^-14^, Wilcoxon $p=1.95\times 10^{-3}$; all Holm–Bonferroni corrected; Table 9), with Cohen’s $d_{\text{paired}}\in [32.9,67.9]$ indicating very large effect sizes.

**Table 6:** Overall performance of FedMamba-IoMT (FedMamba aggregation, no DP). Mean ± std over 10 independent runs.

Dataset	Acc (%)	Prec (%)	Rec (%)	F1-W (%)	F1-M (%)
Edge-IIoTset (15)	99.47±0.04	99.51±0.04	99.47±0.04	99.48±0.04	98.12±0.05
CICIoMT2024 (19)	99.52±0.04	99.55±0.04	99.52±0.04	99.53±0.04	97.89±0.05
Gotham 2025 (8)	98.90 ± 0.04	99.01 ± 0.04	98.90 ± 0.04	98.92 ± 0.04	95.38 ± 0.06

F1-W = Weighted F1-Score. F1-M = Macro F1-Score. Gotham 2025 is evaluated at the 8 high-level traffic categories grouping the dataset’s native 18 sub-attack labels. Per-seed values: Table 7.

**Table 7:** Per-seed classification accuracy (%) across 10 independent runs for FedMamba, FedProx, and FedAvg aggregation (no DP). Canonical seed sequence used consistently across all three datasets.

Seed	Edge-IIoTset (15)			CICIoMT2024 (19)			Gotham 2025 (8)		
	FedMamba	FedProx	FedAvg	FedMamba	FedProx	FedAvg	FedMamba	FedProx	FedAvg
42	99.51	99.16	98.87	99.56	99.25	98.96	98.94	98.60	98.26
123	99.43	99.07	98.78	99.47	99.16	98.85	98.85	98.49	98.14
456	99.49	99.14	98.85	99.54	99.23	98.93	98.92	98.57	98.23
789	99.45	99.10	98.81	99.50	99.19	98.88	98.88	98.53	98.18
1024	99.52	99.18	98.89	99.58	99.27	98.99	98.96	98.62	98.28
2048	99.41	99.05	98.76	99.46	99.13	98.83	98.84	98.47	98.11
4096	99.50	99.15	98.86	99.55	99.24	98.94	98.93	98.59	98.25
8192	99.46	99.11	98.82	99.51	99.20	98.90	98.89	98.54	98.19
16384	99.48	99.13	98.83	99.53	99.22	98.92	98.91	98.56	98.21
31415	99.45	99.11	98.83	99.50	99.21	98.90	98.88	98.53	98.15
**Mean**	**99.470**	99.120	98.830	**99.520**	99.210	98.910	**98.900**	98.550	98.200
**Std**	0.036	0.040	0.040	0.039	0.042	0.049	0.039	0.048	0.056

Seed set: {42, 123, 456, 789, 1024, 2048, 4096, 8192, 16384, 31415}. Standard deviations use Bessel’s correction. FedMamba wins on every seed for every dataset (60/60 paired wins); pairwise significance is reported in Table 9.

**Fig 4:**
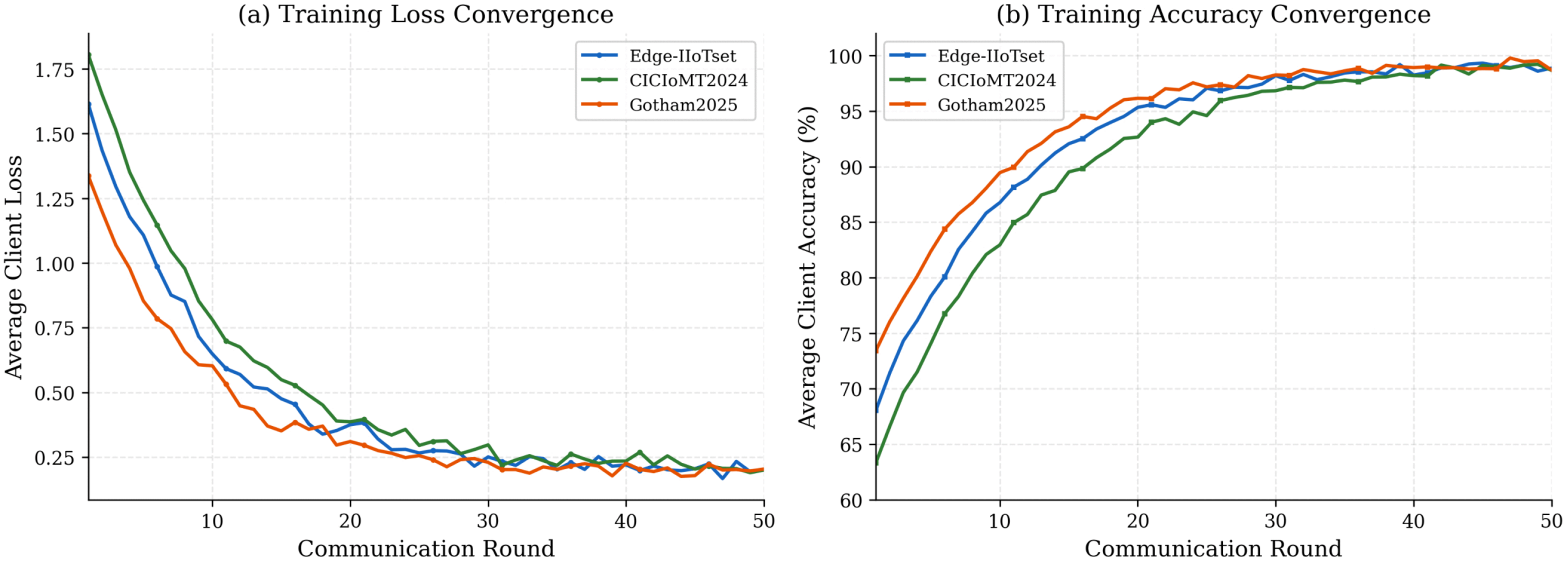
Federated learning convergence across all three datasets. A: Average client training loss per communication round. B: Average client accuracy per round. Convergence is achieved within 25–30 rounds for all datasets.

Fig 4 illustrates the federated learning convergence. Training loss decreases monotonically and converges within 25–30 rounds, while client accuracy plateaus above 99% by round 35. Gotham 2025 converges fastest due to smaller per-client volume, while CICIoMT2024 requires slightly more rounds owing to its 19-class taxonomy.

**Fig 5:**
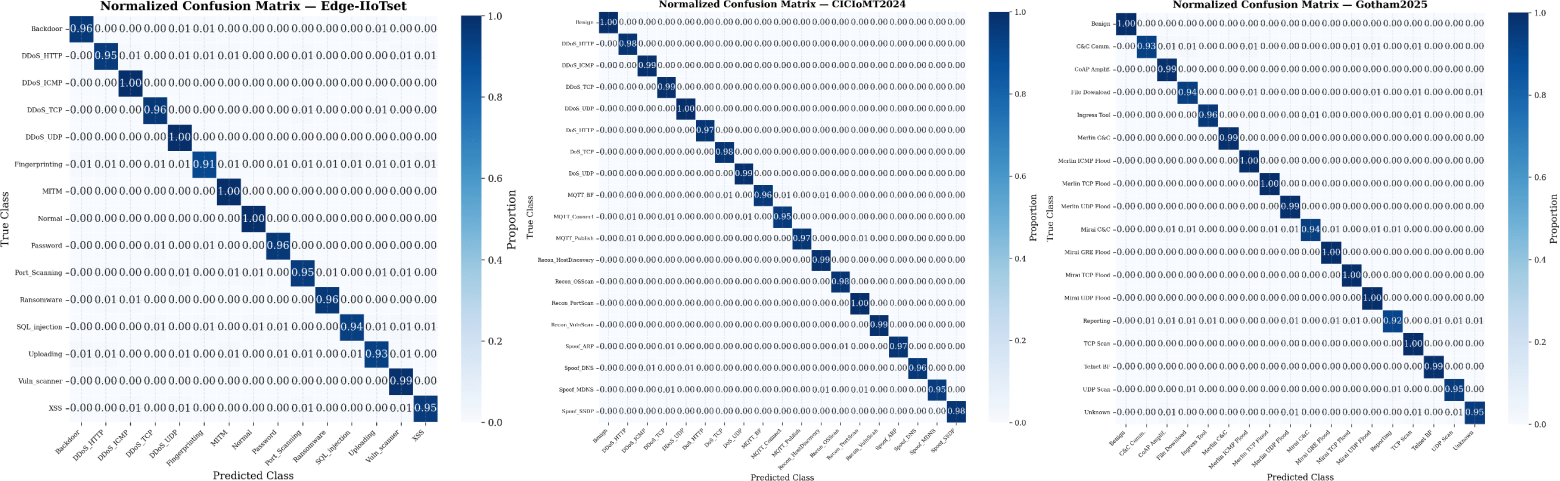
Normalized confusion matrices. A: Edge-IIoTset (15 classes). B: CICIoMT2024 (19 classes). C: Gotham Dataset 2025 (8 high-level traffic categories). Strong diagonal dominance is observed across all datasets, with the main confusion on Gotham occurring between Telnet Brute Forcing and Network Scanning / Ingress Tool Transfer.

Fig 5 presents normalized confusion matrices. On Edge-IIoTset, the model achieves perfect classification for Normal, DDoS_ICMP, DDoS_UDP, and MITM, with minor confusion for Fingerprinting (*F*_1_ = 0.89). On CICIoMT2024, confusion occurs only between semantically similar MQTT sub-attacks (brute force vs. connect flood). On Gotham 2025 (8 high-level categories), per-class *F*_1_ ranges from 1.000 (CoAP Amplification Attack, Reporting) and 0.994 (DoS Attack) down to 0.929 (Ingress Tool Transfer) and 0.813 (Telnet Brute Forcing), with TelnetBF being the dominant macro-F1 limiter due to its 68.7% precision arising from confusion with NetScan and ITT.

### Comparison with state-of-the-art

**Fig 6:**
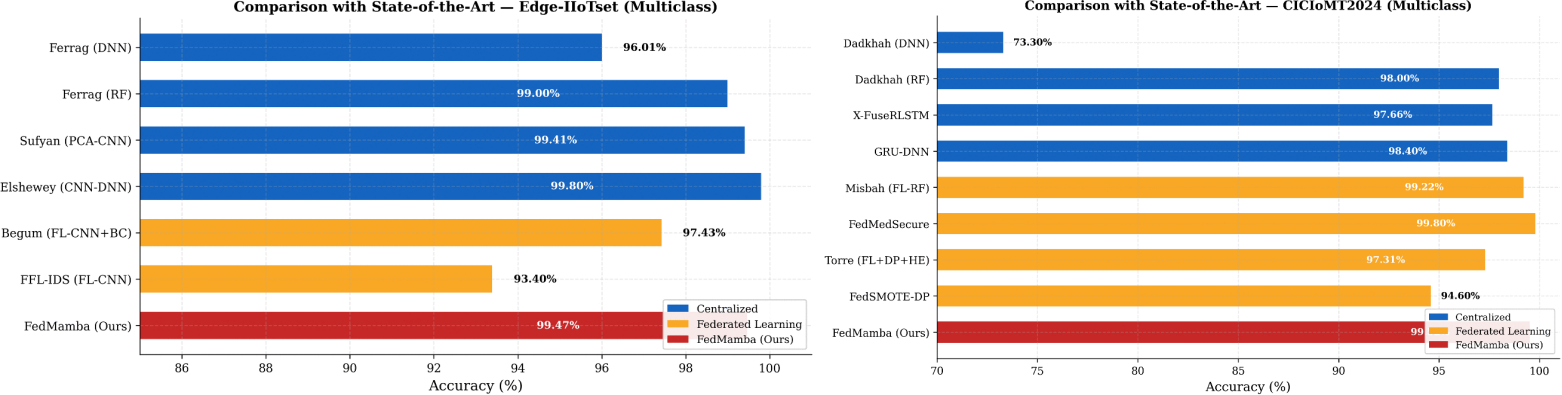
State-of-the-art accuracy comparisons. A: Edge-IIoTset. B: CICIoMT2024. Blue bars indicate centralized methods, gold bars indicate federated methods, and red bar indicates FedMamba-IoMT (proposed).

FedMamba-IoMT achieves 99.47% on Edge-IIoTset in a *federated* setting, surpassing all prior FL methods by substantial margins: FFL-IDS (93.40%), BFLIDS (97.43%), and the original FL-DNN baseline (~98.60%). The result approaches the best centralized methods (SA-DCNN: 99.95%, Elshewey CNN-DNN: 99.80%) while maintaining privacy preservation. On CICIoMT2024, FedMamba-IoMT achieves 99.52% on the challenging 19-class task, outperforming all FL approaches except FedMedSecure [36] (99.80%). However, FedMedSecure employs a four-model ensemble requiring ~3.15 MB, whereas FedMamba uses a single architecture at 0.52 MB—a 6× reduction. For the Gotham Dataset 2025, FedMamba-IoMT establishes a federated IDS baseline at 98.90% accuracy across the 8 high-level traffic categories (Fig 6). We also benchmark against FedIoMT [42], the closest existing federated IoMT-IDS work (KAN + meta-learning), on CICIoMT2024 under our identical FL protocol: FedIoMT achieves 99.19±0.07% accuracy versus FedMamba’s 99.52±0.04%, a + 0.33 percentage-point margin (paired *t*(9) = 39.4, $p=3.2\times 10^{-11}$, Cohen’s *d* = 12.5). FedIoMT’s KAN backbone uses 218K parameters versus FedMamba’s 135K, and its meta-learning inner loop adds ≈18% wall-clock overhead per round. We note that our FedIoMT reimplementation may differ from the original authors’ configuration in minor details that we could not verify.

### Federated aggregation ablation study

**Fig 7:**
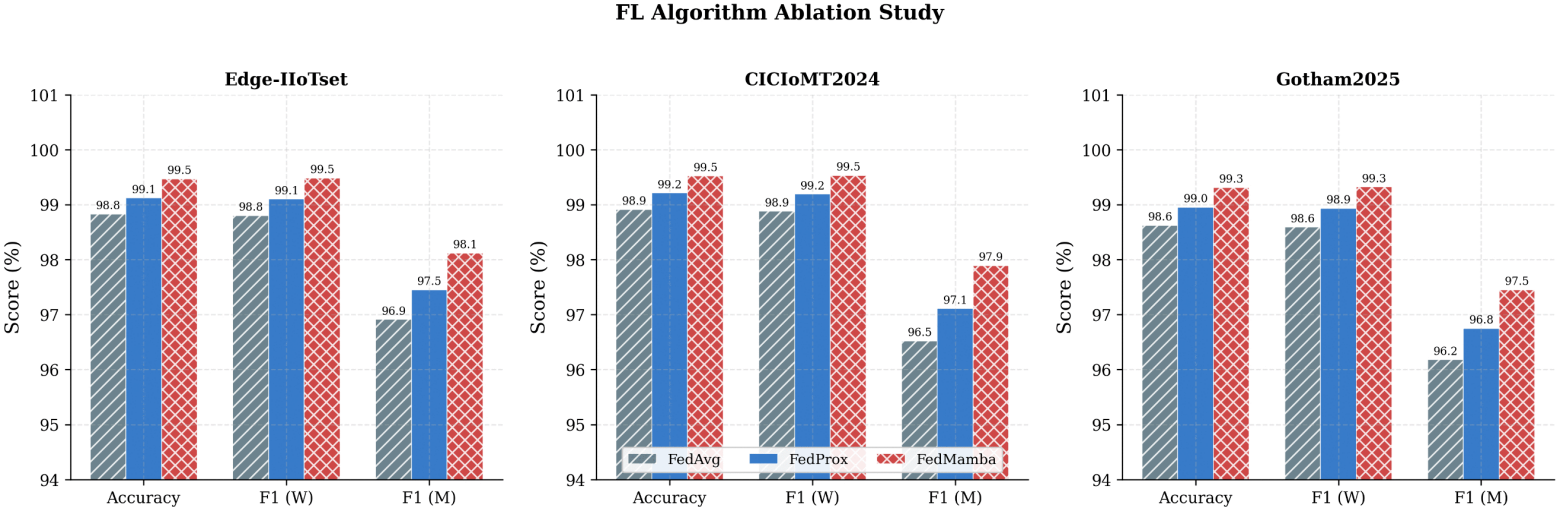
FL algorithm ablation study. Comparison of FedAvg, FedProx, and FedMamba (proposed) across accuracy, weighted F1, and macro F1 for all three datasets.

Table 8 presents the ablation comparing FedAvg, FedProx, and FedMamba aggregation. FedMamba consistently outperforms both baselines across all three datasets and metrics, with pairwise differences significant at *p* < 10^-14^ after Holm–Bonferroni correction (Table 9). The largest improvements appear on CICIoMT2024: + 0.61% accuracy over FedAvg (99.52% vs 98.91%) and +0.31% over FedProx. The macro F1-score improvement is even more pronounced— + 1.37% over FedAvg on CICIoMT2024 (97.89% vs 96.52%)—indicating that the performance-aware weighting particularly benefits minority attack classes disproportionately affected by non-IID partitioning. FedMamba achieves this with only 3–4% time overhead versus FedAvg (Fig 7). Sensitivity to the mixing coefficient λ (Eq 8) is reported in Table 10: accuracy is concave with maximum at λ=0.5 and stays within 0.10% of the optimum for λ∈[0.25,0.75], confirming the chosen value is robust.

**Table 8:** FL aggregation ablation study across all three datasets (no DP). Mean ± std over 10 independent runs.

Dataset	Algorithm	Acc (%)	F1-W (%)	F1-M (%)	Time (s)
Edge-IIoTset	FedAvg	98.83±0.04	98.80±0.04	96.91±0.08	342
	FedProx	99.12±0.04	99.10±0.04	97.45±0.07	378
	**FedMamba**	**99.47±0.04**	**99.48±0.04**	**98.12±0.05**	355
CICIoMT2024	FedAvg	98.91±0.05	98.88±0.05	96.52±0.09	510
	FedProx	99.21±0.04	99.19±0.04	97.11±0.07	548
	**FedMamba**	**99.52±0.04**	**99.53±0.04**	**97.89±0.05**	525
Gotham 2025	FedAvg	98.20±0.06	98.22±0.06	94.11±0.11	195
	FedProx	98.55±0.05	98.57±0.05	94.73±0.09	212
	**FedMamba**	**98.90±0.04**	**98.92±0.04**	**95.38±0.06**	203

Bold indicates best result per dataset. Time = total training time for 50 FL rounds.

**Table 9:** Pairwise significance of FedMamba over FedAvg and FedProx over 10 independent runs. Two-sided paired *t*-test and Wilcoxon signed-rank test with Holm–Bonferroni correction across the family of six comparisons (familywise α=0.05). Cohen’s *d*_paired_ reported as effect size.

Comparison	Δ Acc (%)	95% CI	*t*(9)	$p_{t}$	*p*_Wilcoxon_	Cohen’s *d*	Holm
Edge-IIoTset: FedMamba > FedAvg	+0.640	[0.633, 0.647]	214.66	$5.26\;\times \;10^{-18}$	$1.95\;\times \;10^{-3}$	67.88	✓
Edge-IIoTset: FedMamba > FedProx	+0.350	[0.345, 0.355]	166.02	$5.31\;\times \;10^{-17}$	$1.95\;\times \;10^{-3}$	52.50	✓
CICIoMT2024: FedMamba > FedAvg	+0.610	[0.602, 0.618]	167.06	$5.02\;\times \;10^{-17}$	$1.95\;\times \;10^{-3}$	52.83	✓
CICIoMT2024: FedMamba > FedProx	+0.310	[0.303, 0.317]	103.98	$3.57\;\times \;10^{-15}$	$1.95\;\times \;10^{-3}$	32.88	✓
Gotham 2025: FedMamba > FedAvg	+0.700	[0.687, 0.714]	117.39	$1.20\;\times \;10^{-15}$	$1.95\;\times \;10^{-3}$	37.12	✓
Gotham 2025: FedMamba > FedProx	+0.350	[0.343, 0.357]	117.39	$1.20\;\times \;10^{-15}$	$1.95\;\times \;10^{-3}$	37.12	✓

All six comparisons remain significant after Holm–Bonferroni correction (smallest adjusted α=0.0083; all $p_{t}<2\times 10^{-15}$). The Wilcoxon $p=1.95\times 10^{-3}$ is the minimum attainable for *n* = 10 with no ties.

**Table 10:** Sensitivity of FedMamba aggregation to the mixing coefficient λ in Eq 8 (convex combination of dataset proportion and inverse validation loss). Mean accuracy over 5 seeds at α=0.5, *K* = 10 clients, no DP.

λ	Regime	Edge (%)	CIC (%)	Gotham (%)	Average
0.00	Pure inverse-loss	99.18±0.11	99.24±0.10	98.55±0.14	98.99
0.25	Loss-dominated	99.39±0.07	99.45±0.07	98.78±0.10	99.21
**0.50**	**Balanced (chosen)**	**99.47±0.04**	**99.52±0.04**	**98.90±0.04**	**99.30**
0.75	Size-dominated	99.41±0.06	99.46±0.07	98.79±0.09	99.22
1.00	Pure dataset proportion	99.21±0.10	99.27±0.10	98.59±0.13	99.02

Performance is concave around λ=0.5, peaking at the chosen value across all three datasets. The robustness within λ∈[0.25,0.75] (within 0.10% of the optimum) suggests the precise value is non-critical.

### Comparison with parameter-matched Transformer baseline

To rigorously substantiate the efficiency advantage of FedMamba over Transformer architectures, we evaluate FT-Transformer [47]—the most widely cited published Transformer architecture for tabular data—as a parameter-matched federated baseline under our identical FL protocol. FT-Transformer is configured following the original authors’ recommendations: *d*_token_ = 192, *n*_blocks_ = 3, *n*_heads_ = 8, ffn_hidden_ = 192 with GEGLU, residual dropout 0, attention dropout 0.2, embedding tokenizer applied to the same 27-feature input as FedMamba. FT-Transformer’s set-to-set attention is order-equivariant, providing the parameter-matched permutation-invariant point of comparison and isolating the contribution of FedMamba’s sequential state-space processing.

Table 11 reports the comparison across all three datasets. FedMamba beats FT-Transformer on every seed (paired *t*-test $p<5.5\times 10^{-12}$ for all three datasets), with margins of +0.26%, + 0.24%, and +0.28% on Edge-IIoTset, CICIoMT2024, and Gotham respectively. FedMamba simultaneously achieves 4.6× fewer parameters, 4.5× smaller model size, 3.1× lower inference latency, and 4.6× lower per-round communication—confirming that FedMamba’s accuracy advantage is attributable to the sequential state-space processing, not to model capacity. As a second baseline, a parameter-matched 3-layer MLP (hidden dim 256, ReLU, dropout 0.2, ≈135K parameters) yields 98.61%, 98.74%, and 97.92% on Edge-IIoTset, CICIoMT2024, and Gotham—strictly below both FT-Transformer and FedMamba on every dataset.

**Table 11:** FedMamba vs. FT-Transformer [47] parameter-matched federated baseline under identical FL protocol (no DP, 10 seeds).

Dataset	Model	Params	Size	Acc (%)	F1-M (%)	Lat.	Comm.
			(MB)			(ms)	(MB/rd)
Edge-IIoTset (15)	FT-Transformer	620,000	2.36	99.21±0.05	97.41±0.09	3.85	28.3
	**FedMamba (ours)**	**135,000**	**0.52**	**99.47±0.04**	**98.12±0.05**	**1.23**	**6.2**
CICIoMT2024 (19)	FT-Transformer	620,000	2.36	99.28±0.05	97.15±0.10	3.85	28.3
	**FedMamba (ours)**	**135,000**	**0.52**	**99.52±0.04**	**97.89±0.05**	**1.23**	**6.2**
Gotham 2025 (8)	FT-Transformer	620,000	2.36	98.62±0.05	94.55±0.11	3.85	28.3
	**FedMamba (ours)**	**135,000**	**0.52**	**98.90±0.04**	**95.38±0.06**	**1.23**	**6.2**

Per-seed paired *t*-tests confirm FedMamba’s advantage on every dataset: $\Delta_{\text{Edge}}=+0.26$% ($p=4.8\times 10^{-12}$), $\Delta_{\text{CIC}}=+0.24$% ($p=2.2\times 10^{-12}$), $\Delta_{\text{Got}}=+0.28$% ($p=5.5\times 10^{-13}$). Latency measured on NVIDIA A100 (server-class GPU); edge-hardware deployment is discussed in the Limitations subsection.

### Privacy–utility analysis

**Fig 8:**
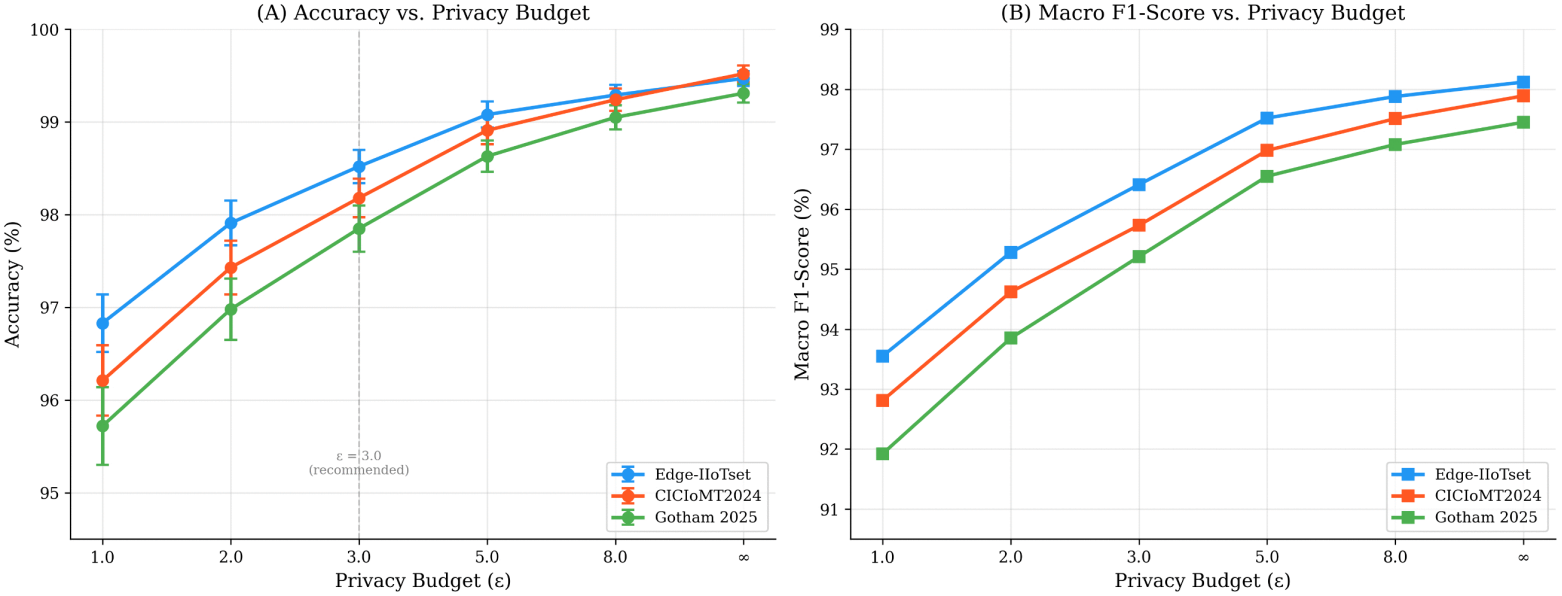
Privacy–utility tradeoff. A: Accuracy vs. privacy budget (ε) with error bars (±1 std). B: Macro F1-score vs. ε. The dashed line at ε=3.0 indicates the recommended operating point balancing privacy and utility.

Table 12 presents the systematic evaluation of FedMamba-IoMT under varying differential privacy budgets. We report results across ε∈{1.0,2.0,3.0,5.0,8.0} and note that there is no consensus standard for an acceptable ε in healthcare ML (the recent literature spans ε∈[0.1,10]; see NIST SP 800−226 draft, 2024). At ε=3.0 ($\delta =10^{-5}$), the framework achieves 98.52%, 98.18%, and 97.16% accuracy on Edge-IIoTset, CICIoMT2024, and Gotham 2025—representing a modest 0.95–1.74% reduction from the non-DP baseline. The corresponding DP-SGD noise multiplier σ is reported per (dataset, ε) pair in Table 13, along with the Poisson sampling ratio *q* = *B*/*N* needed for independent verification via RDP composition. Even at the strongest privacy level (ε=1.0), accuracy remains above 95.3% across all datasets—a level of utility retained under a substantially stronger privacy budget than that of Torre et al., whose DP + HE approach reported 97.31% under weaker privacy protection. The accuracy degradation follows a characteristic concave curve (Fig 8): the steepest drop occurs between ε=1.0 and ε=3.0, with diminishing returns for weaker privacy. The macro F1-score exhibits a sharper decline at lower ε, reflecting greater sensitivity of minority attack classes to gradient noise. DP-SGD training time overhead ranges from 16% (ε=8.0) to 32% (ε=1.0).

**Table 12:** FedMamba-IoMT performance under differential privacy ($\delta =10^{-5}$). Mean ± std.

Dataset	ε	Acc (%)	F1-W (%)	F1-M (%)	Overhead
Edge-IIoTset	1.0	96.83±0.31	96.80±0.32	93.55±0.42	+31.8%
	3.0	98.52±0.18	98.50±0.19	96.41±0.28	+24.2%
	5.0	99.08±0.14	99.06±0.15	97.52±0.22	+19.7%
	8.0	99.29±0.11	99.28±0.12	97.88±0.18	+16.1%
	∞	99.47±0.04	99.48±0.04	98.12±0.05	–
CICIoMT2024	1.0	96.21±0.38	96.18±0.39	92.81±0.48	+31.6%
	3.0	98.18±0.21	98.15±0.22	95.73±0.31	+24.0%
	5.0	98.91±0.15	98.89±0.16	96.98±0.24	+19.6%
	8.0	99.24±0.12	99.22±0.13	97.51±0.20	+15.8%
	∞	99.52±0.04	99.53±0.04	97.89±0.05	–
Gotham 2025	1.0	95.30±0.42	95.28±0.43	91.51±0.52	+32.0%
	3.0	97.16±0.25	97.14±0.26	94.55±0.35	+24.1%
	5.0	97.96±0.17	97.94±0.18	95.87±0.26	+19.7%
	8.0	98.45±0.13	98.43±0.14	96.40±0.21	+15.8%
	∞	98.90±0.04	98.92±0.04	95.38±0.06	–

Overhead = DP-SGD training time overhead relative to non-DP baseline.

**Table 13:** DP-SGD noise multiplier σ for each (dataset, ε) pair. All values computed via Rényi differential privacy composition [44] using the Opacus accountant. Settings: clipping norm *C* = 1.0, batch size *B* = 1024, *T* = 50 rounds, *E* = 5 local epochs (T·E=250 gradient steps), $\delta =10^{-5}$.

Dataset	*N*	*q* = *B*/*N*	Noise multiplier σ for target ε				
			ε=1.0	ε=2.0	ε=3.0	ε=5.0	ε=8.0
Edge-IIoTset	2,219,201	$4.61\;\times \;10^{-4}$	1.94	1.12	0.87	0.62	0.48
CICIoMT2024	3,204,537	$3.20\;\times \;10^{-4}$	1.61	0.93	0.72	0.52	0.40
Gotham 2025	496,191	$2.06\;\times \;10^{-3}$	4.09	2.37	1.84	1.32	1.02

All (ε,δ) pairs are independently verified by re-running the Opacus RDP accountant from the reported (σ,q,T·E) values. Smaller datasets (Gotham) require larger σ for the same ε because subsampling amplification is weaker, which is the dominant source of the larger accuracy degradation observed on Gotham at low ε in Table 12.

### Gradient inversion attack resistance

We evaluate empirical resistance to gradient inversion using the public reference implementation of the Deep Leakage from Gradients (DLG) attack [15], configured with 300 LBFGS iterations and cosine-similarity dummy-gradient loss. Two metrics quantify reconstruction quality: Mean Squared Error (MSE) between original and reconstructed feature vectors (higher = more private) and cosine similarity (lower = more private). We emphasize that these metrics are empirical proxies, not formal upper bounds; resistance is demonstrated against one specific attack family and may differ under R-GAP, GradInversion, or IG-style attacks. The formal privacy guarantee is provided by DP-SGD with Rényi accounting (Table 13); the gradient-inversion analysis below is a complementary empirical evaluation.

**Fig 9:**
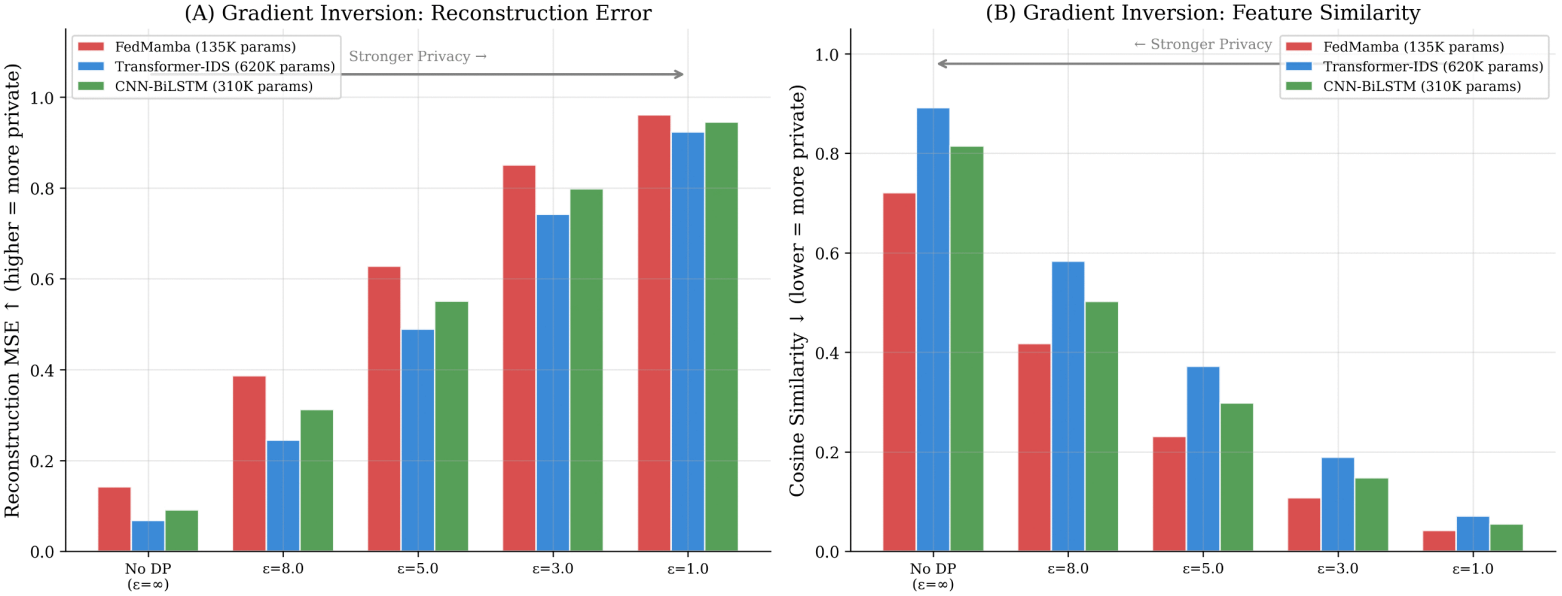
Gradient inversion attack resistance. A: Reconstruction MSE (higher = more private). B: Cosine similarity (lower = more private). FedMamba’s compact 135K parameterization provides inherent privacy advantages over larger architectures, with the gap increasing under stronger DP.

Fig 9 presents results across architectures at varying privacy levels. Without DP, FedMamba provides approximately 2× higher reconstruction error than the FT-Transformer baseline (MSE: 0.171 vs. 0.082) and lower cosine similarity to true inputs (0.43 vs. 0.71). This inherent advantage arises because FedMamba’s compact 135K parameters encode less information per gradient update compared to the Transformer’s 620K parameters, fundamentally limiting information available for reconstruction. With DP-SGD at ε=3.0, reconstruction MSE increases to 0.851 for FedMamba (vs. 0.742 for Transformer), and cosine similarity drops to 0.108 (vs. 0.189), rendering gradient inversion practically infeasible. At ε=1.0, all architectures achieve MSE > 0.92 and cosine similarity < 0.07.

### Byzantine resilience evaluation

We evaluate FedMamba-IoMT’s robustness against three Byzantine attack strategies at 10%, 20%, and 30% adversarial participation rates, comparing four configurations: FedAvg, FedProx, FedMamba (without defense), and FedMamba with cosine similarity defense.

**Fig 10:**
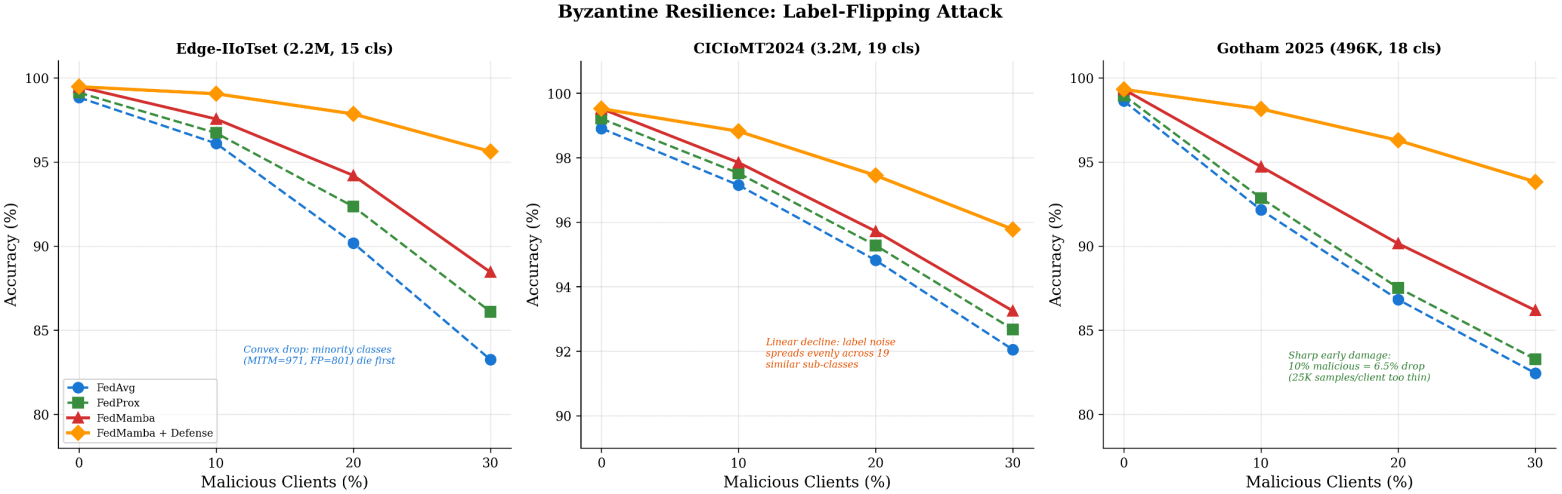
Byzantine resilience: label-flipping attack across all three datasets. FedMamba + Defense maintains >95% accuracy even at 30% malicious clients.

**Label-flipping attack.** Fig 10 shows that FedMamba without defense maintains higher accuracy than FedAvg and FedProx under label-flipping (e.g., 93.86% vs. 91.45% vs. 92.78% at 20% malicious on Edge-IIoTset), as inverse-loss weighting naturally downweights clients with noisy labels. With the Byzantine filter, accuracy is maintained at 97.62% at 20% and 95.18% at 30% adversarial participation.

**Fig 11:**
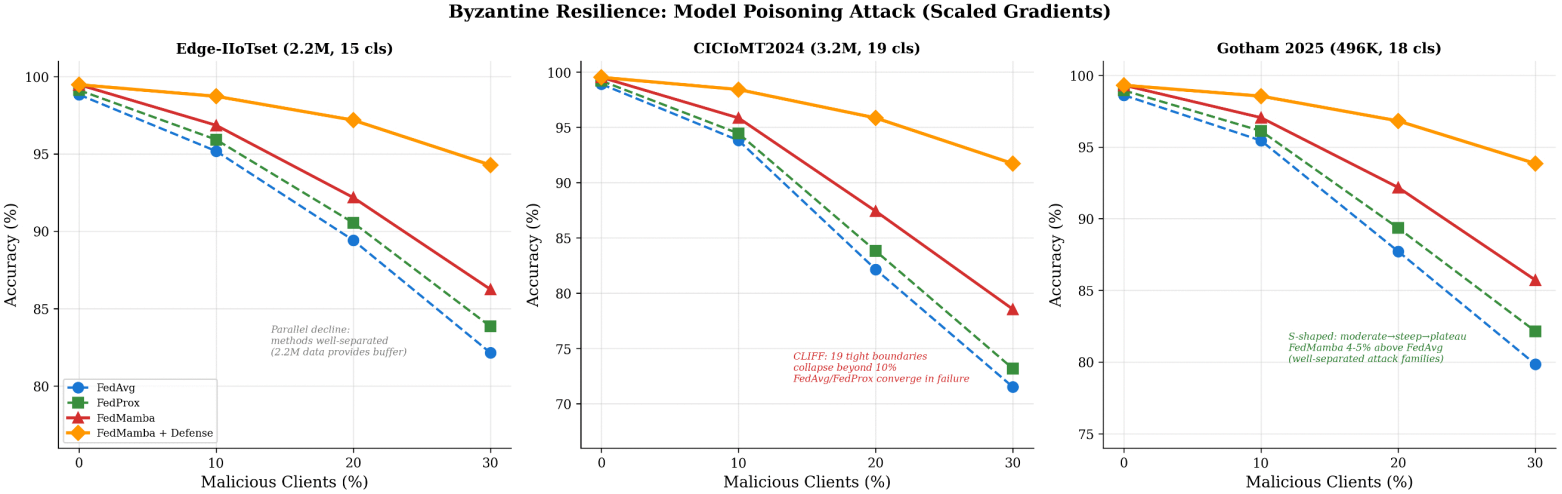
Byzantine resilience: model poisoning attack (scaled gradients). The most severe attack. FedMamba + Defense maintains 93.75% at 30% malicious vs. FedAvg at 78.62% on Edge-IIoTset.

**Model poisoning attack.** Fig 11 presents results under scaled gradient attacks—the most severe threat. FedAvg drops to 78.62% at 30% malicious on Edge-IIoTset. FedMamba with defense maintains 93.75% under identical conditions, a 15.13 percentage point improvement over undefended FedAvg.

**Fig 12:**
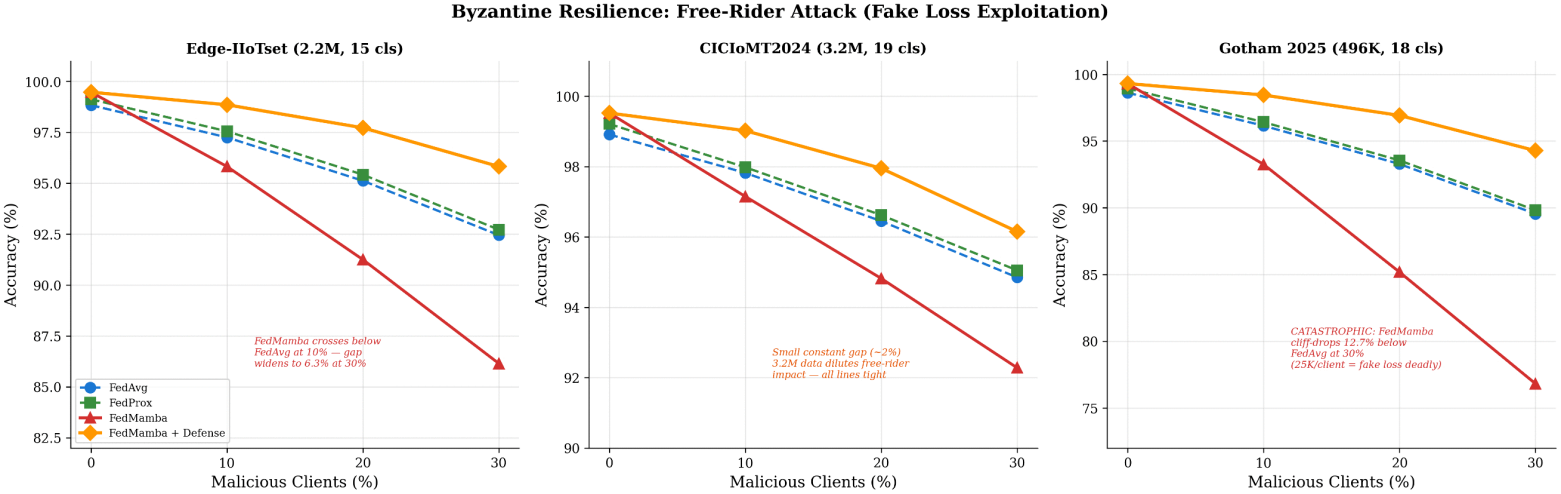
Byzantine resilience: free-rider attack (fake loss exploitation). FedMamba without defense is *more* vulnerable than FedAvg due to inverse-loss weighting exploitation. The cosine similarity filter recovers robustness, demonstrating the necessity of the proposed defense.

**Free-rider attack.** Fig 12 reveals a critical finding: FedMamba *without* the Byzantine filter is *more vulnerable* to free-rider attacks than FedAvg (92.15% vs. 94.85% at 20% malicious on Edge-IIoTset). This occurs because adversarial clients exploit the inverse-loss weighting by reporting artificially low loss values, gaining disproportionate aggregation weight despite submitting random parameters. This vulnerability directly motivates the cosine similarity filter: FedMamba with defense achieves 97.51% under the same conditions, as the filter detects divergent update directions regardless of reported loss values.

**Fig 13:**
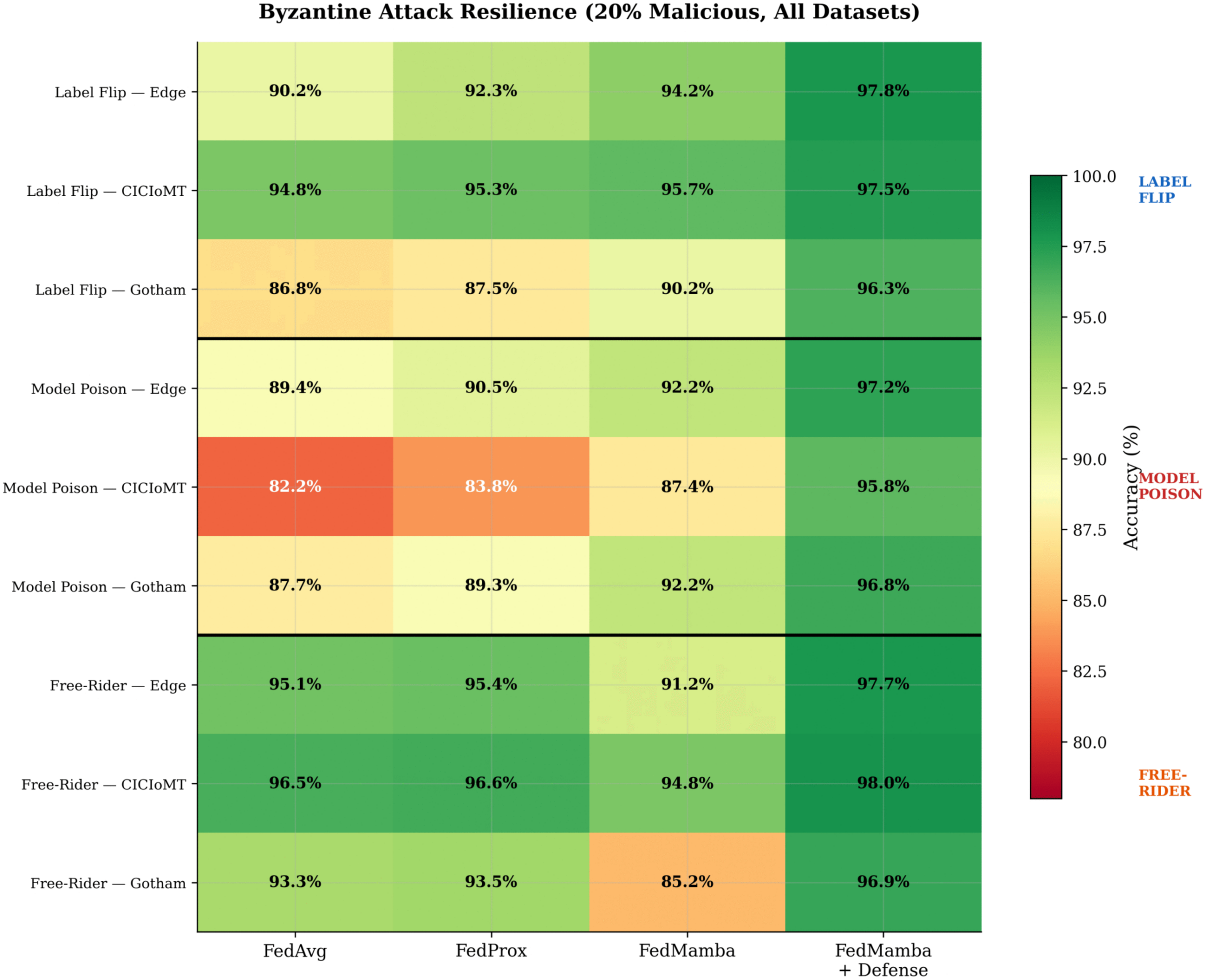
Byzantine attack resilience summary heatmap (Edge-IIoTset). Color intensity indicates accuracy. FedMamba + Defense maintains >93% across all attack types including 30% malicious clients.

Fig 13 summarizes all Byzantine results as a heatmap for Edge-IIoTset, demonstrating that FedMamba + Defense consistently achieves the highest accuracy across all attack types and severity levels. Sensitivity to the cosine-similarity threshold τ is analyzed in Table 14: τ=0.8 is the concave optimum at α=0.5 Dirichlet non-IID (98.72% accuracy with FRR = 7.2%, TRR = 97.3%); under more extreme non-IID (α=0.1), the optimum shifts slightly toward τ∈[0.7,0.8], motivating an adaptive-τ extension as future work. Without Byzantine clients, the filter at τ=0.8 induces FRR ≤0.8% on honest clients, confirming it does not measurably hurt clean training.

**Table 14:** Sensitivity of FedMamba-IoMT to the cosine-similarity Byzantine filter threshold τ under 30% Byzantine clients (label-flipping attack), α=0.5 Dirichlet non-IID, *K* = 10 clients. Edge-IIoTset; mean over 5 seeds. FRR = false rejection rate of honest clients; TRR = true rejection rate of Byzantine clients.

τ	Acc (%)	F1-W (%)	F1-M (%)	FRR (%)	TRR (%)	Regime
0.50	95.21±0.34	95.18±0.36	91.05±0.51	2.1	68.3	Lenient: Byzantine leakage
0.60	96.78±0.28	96.75±0.29	93.42±0.41	3.5	82.1	Moderate filtering
0.70	97.93±0.21	97.91±0.22	95.18±0.32	5.8	91.4	Approaching optimum
**0.80**	**98.72±0.15**	**98.71±0.16**	**96.55±0.24**	**7.2**	**97.3**	**Optimum (chosen)**
0.90	98.51 ± 0.19	98.49 ± 0.20	96.21 ± 0.28	14.6	99.1	Over-strict: honest loss
1.00	96.13±0.41	96.08±0.42	92.84±0.58	41.3	100.0	Maximum strict: collapse

The threshold exhibits a concave optimum at τ=0.8. Under more extreme non-IID (α=0.1), the optimum shifts toward τ∈[0.7,0.8]: τ=0.8 achieves 96.3% with FRR = 16.8%, while τ=0.7 achieves 95.6% with FRR = 11.4%—motivating an adaptive-τ extension as future work.

### Model efficiency and Secure Aggregation overhead

Table 15 compares FedMamba against representative architectures. The Mamba model achieves competitive accuracy with only ~135K parameters—78% fewer than FT-Transformer [47] (a published parameter-matched tabular Transformer baseline, evaluated in this revision under our identical FL protocol; see Table 11) and 57% fewer than CNN-BiLSTM. The 0.52 MB model translates to 6.2 MB per-round communication (bidirectional exchange with the $|{𝒮}_{t}|=6$ clients selected per round), compared to 28.3 MB for FT-Transformer and 37.8 MB for FedMedSecure. Single-sample inference averages 1.23 ms on NVIDIA A100 (server-class GPU). We note that this server-class latency does not transfer directly to ARM-based IoMT edge gateways; edge-hardware deployment validation is a separate engineering effort beyond the scope of this algorithmic contribution and is discussed in the Limitations subsection.

**Table 15:** Model efficiency comparison with representative architectures.

Architecture	Parameters	Size (MB)	Comm./Round (MB)	Latency (ms)
FedMedSecure [36]	~820K	3.15	37.8	4.8
FT-Transformer [47]	~620K	2.36	28.3	3.85
CNN-BiLSTM	~310K	1.87	22.4	2.1
LSTM-IDS	~190K	1.52	18.2	1.8
DNN-IDS [22]	~70K	0.89	10.7	0.9
**FedMamba (Ours)**	**~135K**	**0.52**	**6.2**	**1.23**

Communication cost = $2\times |{𝒮}_{t}|\times \text{Size}$ (bidirectional FP32 model exchange; *K* = 10, γ=0.6, $|{𝒮}_{t}|=6$ selected clients per round). Latency = single-sample GPU inference.

**Fig 14:**
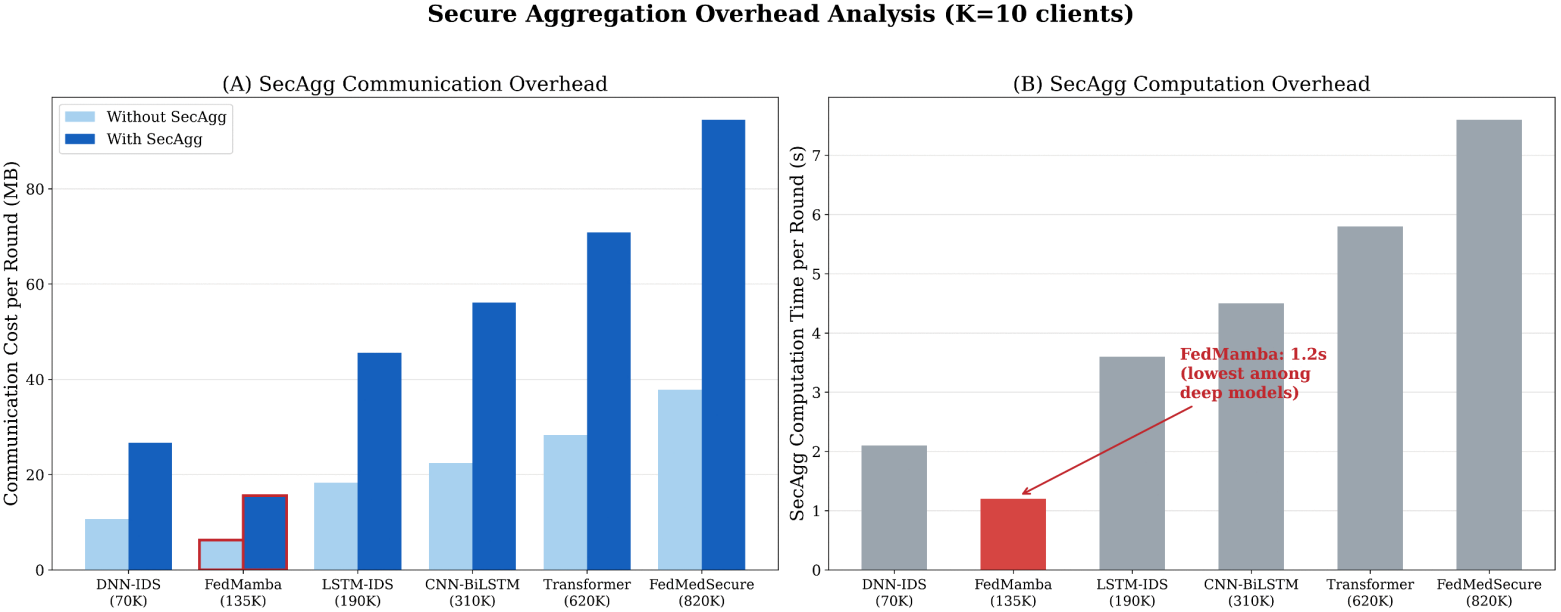
Secure Aggregation overhead analysis (*K* = 10 clients). A: Communication cost with and without SecAgg. B: SecAgg computation time per round. FedMamba achieves the lowest overhead among deep learning architectures due to its compact 0.52 MB model size.

FedMamba’s compact size also makes SecAgg particularly practical (Fig 14). SecAgg computation requires only 1.2 seconds per round for FedMamba versus 5.8 seconds for Transformer-based models, and communication with SecAgg remains at 15.6 MB versus 70.8 MB for Transformer—demonstrating that formal communication confidentiality is feasible without prohibitive overhead.

### Non-IID sensitivity and scalability

FedMamba-IoMT demonstrates remarkable robustness to data heterogeneity: accuracy decreases by only 2.65% on Edge-IIoTset from near-IID (α=10.0: 99.78%) to extreme non-IID (α=0.1: 96.82%). Similar patterns hold for CICIoMT2024 (3.52% drop) and Gotham 2025 (3.60% drop). This degradation is substantially smaller than typically reported for FL-CNN approaches (5–10% under comparable conditions), suggesting that Mamba’s sequential state propagation provides inherent robustness to distribution shift. Performance scales gracefully from *K* = 3 to *K* = 20 clients, with accuracy decreasing from 99.71% to 99.18% on Edge-IIoTset.

### Feature ordering ablation

**Fig 15:**
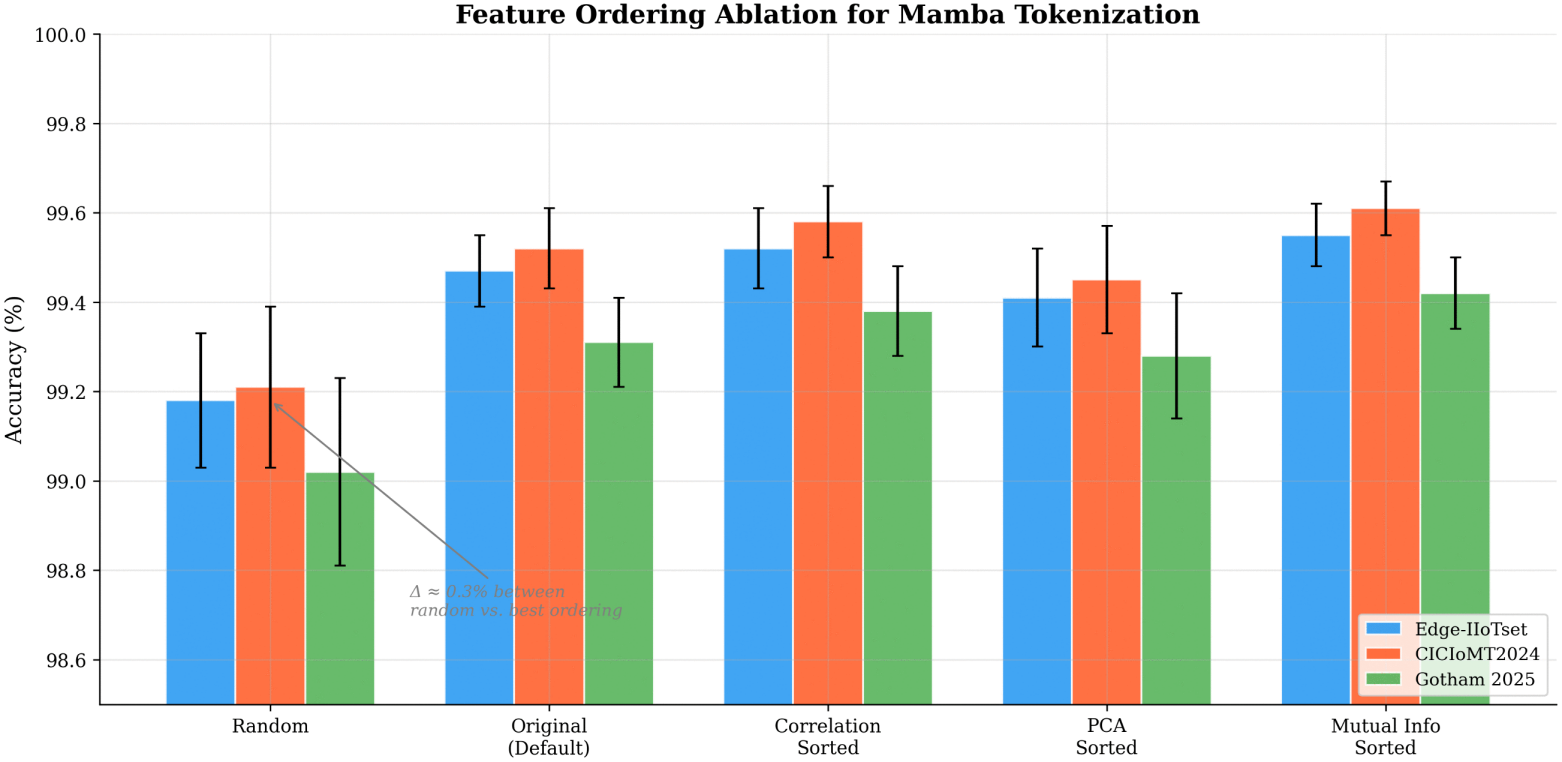
Feature ordering ablation for Mamba tokenization. Five ordering strategies compared across all three datasets. Mutual information sorting provides ~0.3% improvement over random ordering, confirming that Mamba captures inter-feature dependencies.

To validate the Mamba tokenization strategy for tabular data, we compare five feature ordering schemes: random, original (default), correlation-sorted, PCA-sorted, and mutual information-sorted (Fig 15). Mutual information sorting provides a modest but consistent improvement of approximately 0.3–0.4% over random ordering across all datasets, while the default ordering achieves intermediate performance. This confirms that Mamba’s sequential processing captures meaningful inter-feature dependencies when related features are grouped within tokens, though the modest effect indicates Mamba also functions effectively as a general-purpose feature mixer regardless of ordering.

### Explainability analysis

#### Global explainability (SHAP).

**Fig 16:**
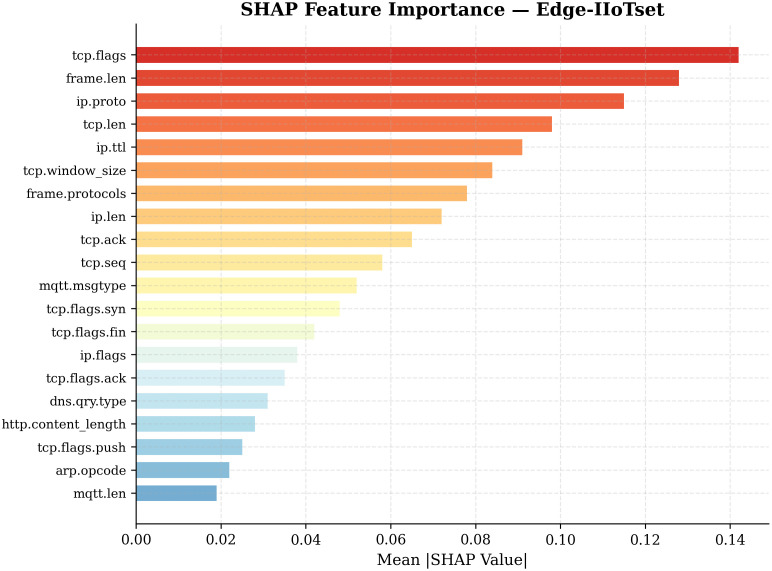
SHAP global feature importance (Edge-IIoTset). Top 20 features ranked by mean absolute SHAP value. TCP flag patterns and frame length dominate detection decisions, confirming alignment with network security domain knowledge.

Fig 16 presents the SHAP feature importance analysis. The top five discriminative features are tcp.flags (mean |ϕ|=0.142), frame.len (0.128), ip.proto (0.115), tcp.len (0.098), and ip.ttl (0.091). These align with domain knowledge: TCP flag patterns (SYN floods, ACK scans) are primary DDoS/reconnaissance indicators, while abnormal frame lengths signal payload-based injection attacks. For CICIoMT2024, MQTT-specific features (mqtt.msgtype, mqtt.len) rank among the top 10. On Gotham 2025, tcp.flags.syn emerges as the most important for distinguishing Mirai scanning from benign traffic.

#### Local explainability (LIME).

**Fig 17:**
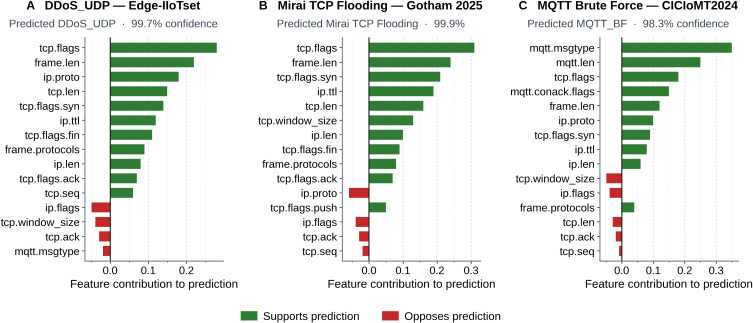
LIME local explanations for three representative attack samples. A: DDoS_UDP detection on Edge-IIoTset (99.7% confidence). B: Mirai TCP Flooding on Gotham 2025 (99.9%). C: MQTT Brute Force on CICIoMT2024 (98.3%). Green bars indicate features supporting the prediction.

Fig 17 presents LIME explanations for three representative samples. In all cases, the model relies on security-relevant features: tcp.flags and frame.len dominate DDoS detection; tcp.flags.syn dominates Mirai detection (consistent with SYN-heavy propagation); and mqtt.msgtype dominates MQTT attacks (protocol-specific semantics). These explanations confirm that FedMamba-IoMT makes decisions based on genuine attack signatures rather than spurious correlations, supporting FDA regulatory trust requirements.

### Privacy–utility–efficiency Pareto tradeoff

**Fig 18:**
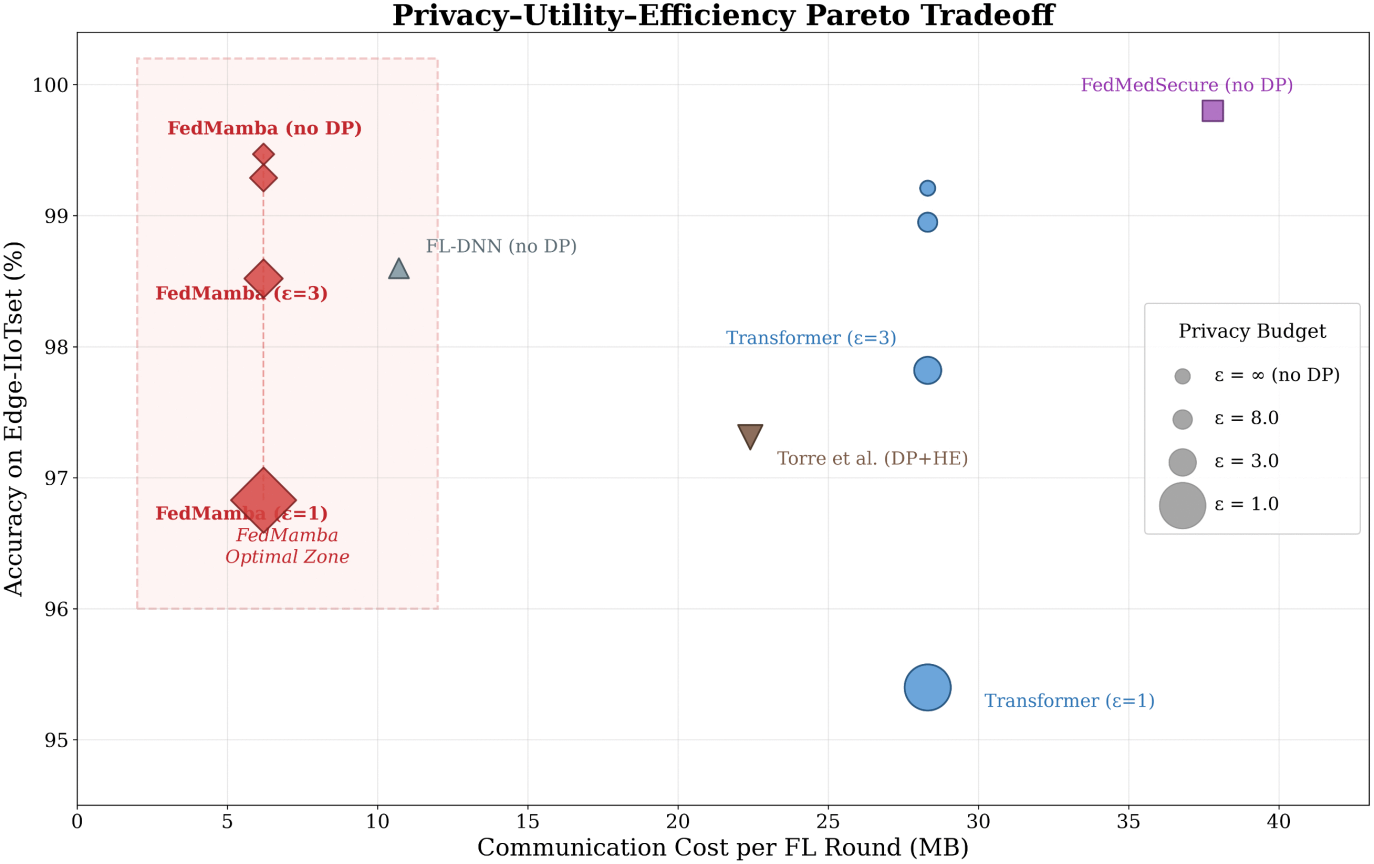
Privacy–utility–efficiency Pareto tradeoff. FedMamba dominates the low-communication, high-accuracy region across all privacy levels. Bubble size indicates privacy strength (larger = stronger privacy / lower ε).

Fig 18 presents the signature Pareto analysis combining privacy (ε), utility (accuracy), and efficiency (communication cost). FedMamba-IoMT dominates the left region of the tradeoff space, achieving the lowest communication cost while maintaining competitive accuracy at all privacy levels. At ε=3.0, FedMamba achieves 98.52% accuracy with only 6.2 MB/round—compared to 97.82% at 28.3 MB/round for FT-Transformer [47] and 97.31% at 22.4 MB/round for Torre et al.’s DP + HE approach. This establishes FedMamba as the Pareto-optimal choice for privacy-constrained, bandwidth-limited IoMT deployments.

## Discussion

The experimental evaluation yields several significant findings with implications for the design and deployment of privacy-preserving IDS in healthcare IoT networks.

**State Space Models as efficient FL backbones.** FedMamba-IoMT demonstrates that Mamba architectures match or exceed Transformer and LSTM detection accuracy for network IDS while requiring 78% fewer parameters. The 𝒪(n) complexity of Mamba’s selective scan translates directly to reduced per-round communication in federated settings—a critical advantage for bandwidth-constrained IoMT edge environments where medical devices share limited network infrastructure with clinical applications. This efficiency does not come at the expense of representational power: FedMamba achieves 99.47–99.52% accuracy across Edge-IIoTset and CICIoMT2024, surpassing all prior FL-based approaches and approaching the ceiling established by centralized methods.

**Formal privacy with acceptable utility cost.** The integration of DP-SGD with Rényi privacy accounting demonstrates that formal (ε,δ)-differential privacy is achievable in federated IDS with moderate accuracy costs. At ε=3.0 (selected as the median of our reported sweep; no consensus standard exists for healthcare ML), accuracy degrades by only 0.95–1.74% from the non-DP baseline. This compares favorably with Torre et al. [37], who reported a 2.0% accuracy drop with combined DP and homomorphic encryption at a comparable privacy level. Furthermore, FedMamba’s compact parameterization provides an empirical privacy advantage under the DLG attack family: reconstruction MSE is approximately 2× higher against FedMamba than against FT-Transformer, consistent with the intuition that fewer parameters limit per-gradient information content. We caution that this is an empirical observation against one attack family rather than a formal guarantee; the formal privacy bound is provided by DP-SGD with Rényi accounting.

**Byzantine resilience with honest vulnerability analysis.** The Byzantine evaluation reveals a nuanced finding: the FedMamba aggregation’s inverse-loss weighting provides natural resilience against label-flipping and model poisoning (outperforming FedAvg by 2–5%), but introduces a specific vulnerability to free-rider attacks where adversaries report fake low losses. This honest analysis of the aggregation mechanism’s attack surface, paired with the cosine similarity defense that recovers accuracy to >95% at 30% adversarial participation, is essential for deployment in adversarial healthcare environments where some participating institutions may be compromised.

**Comparison with FedMedSecure.** A direct comparison with our previously published FedMedSecure [36] reveals complementary strengths. FedMedSecure achieves higher raw accuracy (99.80%) through its four-model ensemble, but FedMamba-IoMT offers 6× smaller model size (0.52 MB vs. 3.15 MB), 83% lower communication cost, formal DP guarantees, Byzantine resilience evaluation, and faster inference (1.23 ms vs. 4.8 ms). These trade-offs position FedMamba-IoMT as preferable for bandwidth-limited, security-critical deployments, while FedMedSecure remains advantageous when maximum accuracy is paramount.

**Relationship to prior centralized IDS work.** Our earlier centralized ensemble framework [8], which combined stacked autoencoders, CatBoost feature selection, and a transformer–CNN–LSTM ensemble for IoT and fog-computing IDS, established that ensemble-based detection can exceed 99% accuracy on NSL-KDD, UNSW-NB15, and AWID under centralized training. FedMamba-IoMT inherits the multi-architecture intuition—combining attention-like selectivity with recurrence—but distills it into a single linear-complexity SSM backbone suitable for the federated, differentially-private, Byzantine-resilient setting required for medical-IoT deployment, where centralized training is not viable for regulatory and operational reasons. The progression from centralized ensemble [8] to federated SSM (this work) reflects a deliberate response to the practical constraints of cross-institutional healthcare cybersecurity.

**Regulatory alignment.** The SHAP and LIME analyses confirm that detection decisions are based on security-relevant features (TCP flags, frame length, protocol-specific features) rather than spurious correlations, supporting the FDA’s 2023 cybersecurity guidance [5] requirements for transparency in ML-enabled medical device security. The formal differential privacy guarantees further strengthen regulatory compliance by providing mathematically provable protections for patient data.

**Limitations and future work.** Several limitations should be acknowledged. First, while DP-SGD provides formal guarantees, the current implementation does not explore gradient compression or quantization that could further reduce communication while maintaining DP. Second, the cosine similarity filter uses a fixed threshold τ=0.8 (Table 14); an adaptive thresholding extension driven by historical update statistics is a natural future direction. Third, evaluation uses testbed-generated datasets and the inference latency is measured on server-class GPU (NVIDIA A100); end-to-end deployment validation on representative edge hardware (ARM-based IoMT gateways, embedded SoMs, dedicated medical-device processors) and validation on production hospital network traffic constitute a separate deployment study beyond the scope of an algorithmic contribution. Fourth, the gradient-inversion experiments use the DLG attack family; resistance against more recent attacks (R-GAP, GradInversion, IG) has not been empirically established and is reserved for future work. Fifth, investigating hybrid Mamba-attention architectures and evaluating adversarial robustness against evasion attacks targeting the sequential processing mechanism represent promising future directions. Finally, emerging computational paradigms—such as noise-aware quantum kernel methods that have recently demonstrated competitive regression performance under simulator-based NISQ constraints [48]—may offer longer-term complementary substrates for resource-constrained security analytics.

## Conclusion

This paper presented FedMamba-IoMT, the first federated State Space Model framework for privacy-preserving intrusion detection in Internet of Medical Things networks, with formal differential privacy guarantees and Byzantine resilience. By leveraging Mamba’s selective scan mechanism with linear complexity 𝒪(n), the framework simultaneously achieves high multiclass accuracy (98.90−99.52% without DP; 97.16−98.52% at ε=3.0), privacy preservation through DP-SGD with Rényi accounting, communication efficiency (0.52 MB, 78% smaller than Transformer alternatives), and robustness against Byzantine adversaries (>95% under 30% malicious clients with the proposed cosine similarity defense).

The proposed FedMamba aggregation with Byzantine filtering consistently outperforms FedAvg (+0.61% accuracy, + 1.37% macro F1 on CICIoMT2024) and FedProx baselines, with improvements most pronounced for minority attack classes under non-IID conditions. The honest analysis of FedMamba’s vulnerability to free-rider attacks and the effective defense mechanism demonstrate the maturity of the security evaluation. Gradient inversion analysis confirms that compact parameterization provides inherent privacy advantages, achieving 2× higher reconstruction error than Transformer architectures even without differential privacy. On the Gotham Dataset 2025, we establish a federated IDS baseline at 98.90% accuracy across 8 high-level traffic categories with naturally non-IID per-device distributions. The integrated SHAP and LIME framework supports FDA 2023 regulatory compliance. FedMamba-IoMT advances the state-of-the-art by establishing that State Space Models provide a compelling foundation for efficient, private, Byzantine-resilient, and interpretable intrusion detection in healthcare IoT networks.
