# Supplementary material for: FedMamba-IoMT: Federated state space models with differential privacy and byzantine resilience for privacy-preserving intrusion detection in Internet of Medical Things
Source: PLoS One. 2026 Aug 10;21(8):e0355601. doi: 10.1371/journal.pone.0355601 (PMC13456290; doi:10.1371/journal.pone.0355601)
Supplement: Supplementary file 1 [file pone.0355601.s002.pdf]

Response to Reviewers

Manuscript PONE-D-26-18683

Title: FedMamba-IoMT: Federated State Space Models with Differential Privacy and Byzantine Resilience for Privacy-Preserving Intrusion Detection in Internet of Medical Things

Journal: PLOS ONE

Corresponding author: Mohammed Tawfik (m.tawfik@su.edu.ye)

Date of submission of revision: 2026-06-05

Dear Dr. Sohail Saif (Academic Editor) and Reviewers,

We thank the Academic Editor and the two Reviewers for the careful and constructive evaluation of our manuscript. The reviewers identified important concerns regarding statistical rigor, baseline definition, threshold sensitivity, privacy reporting, comparative completeness, and reproducibility. We have addressed every comment with new experiments, revised tables, and additional analyses; the manuscript has been substantially strengthened as a result.

Below we reproduce each comment verbatim (italicized) and provide our point-by-point response. All changes are highlighted in the tracked-changes version of the manuscript. New tables introduced in this revision are referenced as Table R# (matching the comment number). All new experiments use the canonical 10-seed sequence {42, 123, 456, 789, 1024, 2048, 4096, 8192, 16384, 31415}.

Summary of major changes

- Re-ran all experiments with 10 independent seeds (up from 3). Means are essentially unchanged.
- Clarified the Gotham 2025 evaluation taxonomy: the dataset's native 18 fine-grained sub-attack labels group into 8 high-level attack categories (per the dataset authors' own taxonomy); we evaluate at the 8-category level (Benign, CoAP Amplification Attack, DoS Attack, Ingress Tool Transfer, Network Scanning, Periodic C&C Communication, Reporting, Telnet Brute Forcing), which matches the confusion-matrix figure and avoids data-sparsity in long-tail Mirai sub-variants. Reported accuracy at this level is 98.90%.
- Added paired two-sided t-tests and Wilcoxon signed-rank tests with Holm–Bonferroni correction for all pairwise comparisons of FedMamba vs. FedAvg and FedMamba vs. FedProx (new Table R-Sig).
- Replaced the undefined "Transformer-IDS" baseline with FT-Transformer (Gorishniy et al., NeurIPS 2021) — a published, citable, parameter-matched tabular transformer evaluated under the identical FL protocol (new Table R-FT).
- Added τ-threshold sensitivity analysis with explicit false-rejection-rate reporting (new Table R-τ).
- Reported the noise multiplier σ for every (dataset, ε) pair, with subsampling ratio q (new Table R-DP). Removed the unsupported claim that $\varepsilon = 3.0$ is a "medical-application standard."
- Added λ-sensitivity ablation for the FedMamba aggregation mixing coefficient (new Table R-λ).
- Added direct comparison with FedIoMT (Fahim-Ul-Islam et al., 2025) on CICIoMT2024.
- Added Gotham per-device native partitioning experiment (78 devices as 78 federated clients).
- Softened all gradient-inversion claims and re-ran experiments using the public DLG implementation (Zhu et al., 2019) for reproducibility.

- Added a paragraph in the Limitations subsection acknowledging that edge-hardware deployment and clinical-network validation are beyond the scope of this algorithmic contribution, in response to the corresponding reviewer comments.

Journal requirements (Academic Editor)

Requirement 1 — PLOS ONE style and file naming

> *Please ensure that your manuscript meets PLOS ONE's style requirements, including those for file naming.*

The manuscript has been reformatted using the PLOS ONE LaTeX templates (formatting_sample_main_body.pdf and formatting_sample_title_authors_affiliations.pdf). All figure files have been renamed to follow the "Fig 1", "Fig 2", "Fig 3" convention used in the in-text citations. The main_no_figs.tex source has been cleaned and a separate .bib file is provided.

Requirement 2 — Code sharing

> *PLOS ONE has specific guidelines on code sharing for submissions in which author-generated code underpins the findings.*

All code used to generate the reported results — including the FedMamba-IoMT model, DP-SGD pipeline with RDP accounting, Byzantine-resilient aggregation with the cosine-similarity filter, SHAP/LIME explainability hooks, and the experiment scripts that produced every table in the manuscript — has been released under the MIT license at https://github.com/kmkholm/FedMamba-IoMT. The repository contains the canonical seed configuration, environment specification (Python 3.11, PyTorch 2.1, Opacus 1.4.0), random-seed-reproducible launch scripts for every reported experiment, and unit tests for the privacy accountant. The release will be made public upon acceptance; an anonymous preview link has been provided to the editorial office.

Requirement 3 — Figure 1 copyright

> *We note that Figure 1 in your submission contains images which may be copyrighted.*

Figure 1 (overview of the IoMT threat model) has been completely redrawn by the authors using original vector graphics. The revised Figure 1 contains no third-party images; all device icons are either authored from scratch or sourced from CC0 / public-domain repositories (Font Awesome under SIL OFL/CC-BY 4.0 and Material Icons under Apache 2.0). Attribution lines have been added to the caption per the requested CC-BY 4.0 licensing terms.

Requirement 4 — Figure file naming

> *We note that while your manuscript text cites 18 main figures and have captions included, several of your uploaded figure items use descriptive text names rather than specific figure numbers.*

All figure files have been renamed in sequential order: Fig1.png, Fig2.png, Fig3.png, …, Fig18.png. The in-text citations and figure captions have been updated to use the matching \ref{} labels (fig1, fig2, etc.). A figure-file map is provided as supplementary information.

Reviewer #1

We thank Reviewer #1 for the exceptionally thorough and rigorous evaluation. The ten major comments below have driven the most substantive improvements to the manuscript.

Major Comment 1 — Statistical validity (3 seeds)

> *All reported results are based on only three random seeds {42, 123, 456}. The primary claims of superiority over FedAvg and FedProx rest on accuracy margins of 0.36–0.64%, and the claims of state-of-the-art federated performance rest on margins of 0.08–0.10% standard deviation ranges. With only three experimental runs, these margins cannot be statistically distinguished from noise. ... Required action: increase the number of independent runs to a minimum of five, and preferably ten, and report statistical significance tests for all primary pairwise comparisons.*

Response.

We re-ran every primary experiment with 10 independent seeds (the canonical sequence {42, 123, 456, 789, 1024, 2048, 4096, 8192, 16384, 31415}) and added formal hypothesis testing. The new Table 3 (overall performance), Table 6 (aggregation ablation), and the new Tables R1 (per-seed accuracies) and R-Sig (significance tests) reflect this. Key findings:

- Means are essentially unchanged from the 3-seed reporting. Edge-IIoTset 99.47% and CICIoMT2024 99.52% are identical. Gotham 2025 is reported at 98.90% on the 8-category evaluation (the high-level grouping of the dataset's 18 sub-attack labels), consistent with the confusion-matrix figure.
- Standard deviations tighten approximately as $1/\sqrt{n}$ (≈0.04% for n=10 vs. ≈0.10% for n=3).
- FedMamba beats both FedAvg and FedProx on every single seed for every dataset (60/60 paired wins).
- Paired two-sided t-tests: $p < 1.2 \times 10^{-15}$ for every comparison. Wilcoxon signed-rank: $p = 1.95 \times 10^{-3}$ (the minimum value attainable for n=10 with all paired differences in one direction).
- Holm-Bonferroni correction over the family of 6 comparisons (3 datasets × 2 baselines): all 6 remain significant at the smallest adjusted $\alpha = 0.0083$.
- Cohen's d_paired ranges from 32.9 to 67.9, well above the 0.8 threshold for large effects.

Power analysis: at the observed effect sizes and the new n=10, post-hoc statistical power (G*Power, paired-samples t) exceeds 0.999 for every comparison. This addresses the concern that small n could mask noise; the effect sizes are large enough that 3 seeds would also have detected them, and the increased n now provides rigorous confirmation.

Manuscript changes: Last sentence of "Data preprocessing and implementation details" updated to specify 10 seeds and the new significance protocol. Revised Table 3 and Table 6. New Tables R1 (per-seed) and R-Sig (significance) added. First paragraph of "Overall classification performance" rewritten.

Major Comment 2 — Undefined Transformer-IDS baseline

> *The paper's central efficiency claims ... all rely on a "Transformer-IDS" with approximately 620K parameters that appears in Table 8 and throughout the results. However, this model is never defined. There is no citation for this architecture, no description of the number of attention heads, layers, feedforward dimensions, or training configuration ... Required action: provide full architectural specifications and a citation or open-source implementation, or replace this undefined baseline with a published, citable Transformer-based IDS model evaluated under identical conditions.*

Response.

We replaced the undefined "Transformer-IDS" entirely with FT-Transformer (Gorishniy et al., NeurIPS 2021, "Revisiting Deep Learning Models for Tabular Data"), the most widely cited published Transformer architecture for tabular data. The replacement provides:

- Citable architecture (NeurIPS 2021; >800 citations; reference implementation on github.com/yandex-research/rtdl).
- Full hyperparameter specification (now in new Table R-FT caption): d_token=192, n_blocks=3, n_heads=8, ffn_hidden=192 with GEGLU, residual dropout 0, attention dropout 0.2, embedding tokenizer applied to the same 27-feature input. All FL hyperparameters (T=50 rounds, E=5 epochs, K=10 clients, $\eta=10^{-3}$, batch 1024) are identical to FedMamba's protocol.
- Federated accuracy on every dataset: 99.21% on Edge-IIoTset, 99.28% on CICIoMT2024, 98.62% on Gotham 2025.
- Parameter count: 620,000 (≈4.6× FedMamba's 135K).

New Table R-FT provides the full head-to-head comparison. FedMamba beats FT-Transformer on every seed for every dataset (paired t-test $p < 5.5 \times 10^{-12}$, all Holm-Bonferroni corrected). The 78% parameter reduction, 78% communication reduction, and 3.1× inference-speed advantage of FedMamba over FT-Transformer are now rigorously substantiated against a published, reproducible baseline.

Manuscript changes: All references to "Transformer-IDS" replaced with "FT-Transformer" with citation [gorishniy2021revisiting]. New Table R-FT added. Table 8 efficiency comparison reworked to use FT-Transformer values throughout. Gradient-inversion comparison (Major 7) also reworked to use FT-Transformer.

Major Comment 3 — No direct comparison with FedIoMT

> *The authors identify FedIoMT (Fahim-Ul-Islam et al., 2025) as "the closest existing work to FedMamba-IoMT in concept" ... Despite this acknowledgment, no direct quantitative comparison between FedMamba-IoMT and FedIoMT is provided on any shared dataset. Required action: include a direct experimental comparison against FedIoMT on at least one shared benchmark dataset, under equivalent or clearly described federated conditions.*

Response.

We implemented FedIoMT (KAN + meta-learning federated IDS) following the architecture and training protocol described in Fahim-Ul-Islam et al. (2025), and evaluated it on the shared CICIoMT2024 benchmark under our identical federated conditions (K=10 clients, $\alpha=0.5$ Dirichlet non-IID, T=50 rounds, E=5 epochs). FedIoMT achieves 99.19 ± 0.07% accuracy on CICIoMT2024 vs. FedMamba's 99.52 ± 0.04% — a margin of +0.33 percentage points (paired t-test $t(9) = 39.4$, $p = 3.2 \times 10^{-11}$, Cohen's $d = 12.5$, all Holm-Bonferroni corrected). FedIoMT's KAN backbone uses 218K parameters vs. FedMamba's 135K, and its meta-learning inner loop adds ≈18% wall-clock overhead per round. Macro F1 also favors FedMamba: 97.89% vs. 97.41%, particularly on the rare MQTT brute-force class.

We were unable to reproduce FedIoMT's reported results on Edge-IIoTset or Gotham (the original authors did not release weights or per-seed scores for those datasets), and our reimplementation may differ in minor implementation details that we could not verify. We discuss this limitation explicitly in the revised "Comparison with state-of-the-art" subsection.

Manuscript changes: New paragraph in "Comparison with state-of-the-art" subsection presenting the FedIoMT comparison. New row added to Table 7 (SOTA comparison) for CICIoMT2024. Honest discussion of reproducibility limitations added.

Major Comment 4 — τ threshold sensitivity and false rejection rate

> *The cosine similarity threshold τ = 0.8 is a critical hyperparameter ... no theoretical justification ... no empirical sensitivity analysis across alternative values, and no analysis of the false rejection rate (legitimate clients incorrectly filtered) at this threshold under varying degrees of data heterogeneity. Required action: provide a sensitivity analysis of model performance across a range of τ values (e.g., τ ∈ {0.5, 0.6, 0.7, 0.8, 0.9}) under at least one dataset and α = 0.5. Additionally, the false rejection rate for honest clients must be reported at τ = 0.8.*

Response.

We added a full sensitivity sweep across τ ∈ {0.5, 0.6, 0.7, 0.8, 0.9, 1.0} on Edge-IIoTset under 30% Byzantine label-flipping with α = 0.5 Dirichlet non-IID, plus a secondary sweep at α = 0.1 (extreme non-IID). The new Table R-τ reports for each τ: accuracy, weighted/macro F1, false rejection rate (FRR) on honest clients, and true rejection rate (TRR) on Byzantine clients.

- τ = 0.8 is the concave optimum at α = 0.5: 98.72% accuracy with FRR = 7.2% and TRR = 97.3%.
- τ = 1.0 collapses to 96.13% with FRR = 41.3% — the strict threshold rejects too many honest updates.
- τ ≤ 0.6 leaks Byzantine updates and degrades accuracy (TRR ≤ 82%).
- Under α = 0.1 (extreme non-IID), the optimum shifts slightly toward τ ∈ [0.7, 0.8] because honest clients diverge more naturally; we note this in the manuscript and propose an adaptive-τ extension as future work.
- Without Byzantine clients, the filter at τ = 0.8 induces FRR ≤ 0.8% on honest clients, confirming it does not measurably hurt clean training.

A theoretical analysis of the threshold is included in the revised "Byzantine resilience" subsection: under the assumption that honest gradients are drawn from a sub-Gaussian distribution with cosine deviation bounded by σ_cos, and Byzantine gradients have expected cosine similarity ≤ μ_byz < τ, the per-round error in the aggregated update is bounded by O(σ_cos · √log K) with high probability. The bound is tightest near τ ≈ E[cos_honest] − σ_cos · √log K, which for our setting matches τ ≈ 0.8 empirically.

Manuscript changes: New Table R-τ added. "Byzantine resilience" subsection extended with the sensitivity sweep, theoretical sketch, and adaptive-τ discussion.

Major Comment 5 — DP noise multiplier and ε=3.0 "medical standard" claim

> *The paper claims formal (ε, δ)-differential privacy guarantees ... However, the noise multiplier σ — the parameter that actually determines the privacy-utility trade-off and is required for independent verification of the claimed ε values — is never reported for any of the five privacy budgets. Without knowing σ, C, and the batch sampling ratio q for each dataset, it is impossible for reviewers or readers to verify that the claimed ε values are correctly computed. Furthermore, the paper states that ε = 3.0 is "a privacy budget widely considered suitable for medical applications," citing Abadi et al. (2016). This citation ... misrepresents the state of the field.*

Response.

We added a dedicated noise-multiplier table (new Table R-DP) reporting σ for every (dataset, ε) pair, along with the sampling ratio $q = B/N$ and the fixed clipping norm $C = 1.0$, batch size $B = 1024$, and step count $T \cdot E = 250$. All σ values are computed via the Opacus RDP accountant (mironov2017renyi, wang2019subsampled) and independently verified by re-running the privacy accountant from $\sigma \rightarrow \varepsilon$. Smaller datasets (Gotham, $q = 2.06 \times 10^{-3}$) require larger σ ($\sigma = 1.84$ at $\varepsilon = 3.0$) than larger datasets (Edge-IIoTset, $q = 4.61 \times 10^{-4}$, $\sigma = 0.87$ at $\varepsilon = 3.0$) for the same ε because subsampling amplification is weaker — this is also the dominant cause of Gotham's larger accuracy degradation at low ε observed in Table 7.

On the ε-as-medical-standard claim: we removed the assertion that $\varepsilon = 3.0$ is "widely considered suitable for medical applications." The Abadi et al. (2016) citation supports DP-SGD as the mechanism but not any consensus on ε ranges in healthcare. The revised text now states that no such consensus exists, surveys the actual literature range ($\varepsilon \in [0.1, 10]$ for healthcare ML in recent works), references NIST SP 800-226 (draft, 2024) for current US guidance, and presents the full sweep $\varepsilon \in \{1, 2, 3, 5, 8\}$ for practitioners to select from based on their specific threat model and regulatory regime.

Manuscript changes: New Table R-DP added. "Privacy-utility analysis" subsection rewritten: the "medical-standard" sentence removed, replaced with an honest survey of healthcare-ML ε values in the literature and a reference to NIST SP 800-226.

Major Comment 6 — Permutation-invariant tabular baseline

> *The paper does not compare FedMamba against any permutation-invariant tabular baseline such as a standard MLP, TabNet, or FT-Transformer with matched parameter counts under identical federated conditions. Without such a comparison, it cannot be determined whether the Mamba sequential processing is actually contributing to performance or whether the results are achievable with a simpler architecture.*

Response.

The FT-Transformer baseline introduced in our response to Major Comment 2 directly addresses this. FT-Transformer is a permutation-invariant tabular Transformer (its set-to-set attention is order-equivariant; positional encoding is intentionally absent in the standard formulation). At 620K parameters — 4.6× FedMamba's 135K — it provides the parameter-matched permutation-invariant point of comparison the reviewer requests. FedMamba's accuracy advantage of +0.24–0.28% on every dataset (Table R-FT) is therefore directly attributable to the sequential state-space processing, not to model capacity or general architectural complexity.

Additionally, we ran a standard 3-layer MLP baseline (matched at 135K parameters via hidden dim 256, ReLU, dropout 0.2) under the identical FL protocol. Federated accuracy: 98.61% Edge, 98.74% CIC, 97.92% Gotham — strictly below both FT-Transformer and FedMamba on every dataset, confirming that the gap is not closed by either a larger permutation-invariant model (FT-Transformer) or a parameter-matched permutation-invariant model (MLP). These results are now in the revised Table 8 (efficiency-vs-accuracy).

Manuscript changes: FT-Transformer (per Major 2) plus parameter-matched MLP baseline both added to Table 8 and discussed in the "Comparison with parameter-matched Transformer baseline" subsection.

Major Comment 7 — Gradient inversion: self-implemented attack

> *The gradient inversion attack used for this evaluation is a self-implemented simulation against the authors' own undefined Transformer baseline. No published gradient inversion attack implementation … is used. … Required action: use a published, reproducible gradient inversion attack implementation … or substantially soften their language regarding gradient inversion resistance.*

Response.

We adopted both recommendations. First, we re-ran the gradient inversion experiment using the public reference implementation of DLG (Deep Leakage from Gradients; Zhu et al., NeurIPS 2019) from github.com/mit-han-lab/dlg, against the FT-Transformer baseline (Major 2) and FedMamba under matched conditions. Attack configuration: 300 LBFGS iterations, dummy-gradient cosine-similarity loss, identical victim batch (size 32 from CICIoMT2024), no DP. Results:

- FT-Transformer (620K params): reconstruction MSE = 0.082, cosine sim to true input = 0.71.
- FedMamba (135K params): reconstruction MSE = 0.171, cosine sim to true input = 0.43.
- FedMamba achieves 2.08× higher reconstruction MSE — consistent with the previously reported 2× claim, now using a published attack.

Second, we substantially softened the language. The revised "Gradient inversion analysis" subsection now states that (a) MSE and cosine similarity are proxies, not formal upper bounds; (b) the resistance is empirical against one specific attack family; (c) modern gradient inversion attacks (R-GAP, GradInversion, IG) may achieve different results on small models, and we cannot claim general resistance; (d) the integration with DP-SGD (Table R-DP) provides the formal privacy guarantee.

Manuscript changes: Gradient inversion experiment re-run with DLG (Zhu et al., 2019); reference and DOI added. Language softened throughout the "Gradient inversion analysis" subsection. Figure 9 updated with new MSE/cosine values.

Major Comment 8 — Gotham native per-device partitioning unused

> *The Gotham Dataset 2025 … uses K=10 clients with Dirichlet partitioning, discarding the dataset's native per-device partitioning structure. The primary advantage of this dataset … is therefore not utilized.*

Response.

We added a complete experiment using the native per-device partitioning. The 78 emulated devices in Gotham 2025 are each assigned as one federated client (K = 78), and the global model is trained for T = 50 rounds with E = 5 local epochs and $\eta = 10^{-3}$ as before. Sub-sampling each round selects 10 random devices (≈13% participation, matching realistic IoMT deployment). Under this native partitioning:

- FedMamba accuracy: 98.71 ± 0.06% (vs. 98.90% under the Dirichlet K=10 partitioning) — a small 0.19% drop due to higher per-device heterogeneity.
- FedAvg accuracy: 97.84 ± 0.11% (vs. 98.20% under Dirichlet) — a larger 0.36% drop, demonstrating that FedMamba's loss-aware weighting is more robust under realistic per-device skew.
- Macro F1 (FedMamba): 94.93% (vs. 95.38% under Dirichlet) — the long-tail Telnet Brute Forcing class remains the dominant difficulty.
- Per-round communication scales as $O(K\_active \times |\theta|)$; for K_active = 10 this matches our existing reporting at 41.6 MB/round.

These results confirm that FedMamba's advantage holds — and in fact widens — under the ecologically valid per-device partitioning the dataset was designed for. The new experiment is reported in a dedicated subsection "Gotham native per-device evaluation."

Manuscript changes: New subsection "Gotham native per-device evaluation" added under Results. Per-device experiment summary table added (Table R-Got).

Major Comment 9 — A100 inference latency claim

> *The 1.23ms inference latency is measured on an NVIDIA A100 GPU. IoMT edge gateways and medical device processors are typically ARM-based embedded systems with orders-of-magnitude less compute. The claim that FedMamba enables "real-time detection" based on A100 measurements is not transferable to the stated deployment context.*

Response.

We agree with the reviewer's point. The contribution of this paper is the FedMamba-IoMT algorithmic framework — its architecture, federated aggregation, privacy mechanism, and Byzantine resilience — evaluated on three large-scale benchmarks. End-to-end deployment validation on representative edge hardware (ARM-based gateways, embedded SoMs, dedicated medical-device processors) is a separate engineering effort that depends on the target deployment, quantization strategy, and runtime, and is beyond the scope of an algorithmic contribution. We have therefore made two specific changes:

- All references to "real-time detection" have been qualified throughout the manuscript. The latency number is now consistently reported as "1.23 ms on NVIDIA A100 (server-class GPU)", with no claim of transferability to edge hardware.
- The Limitations subsection has been extended with an explicit paragraph stating that edge-hardware latency on ARM-based IoMT gateways is unmeasured in this work and is a necessary step for deployment validation in future work. We note that FedMamba's compact 0.52 MB model size and linear-complexity inference are favorable for edge deployment in principle, but empirical edge measurements are not claimed in this revision.

Manuscript changes: "Real-time" language qualified throughout. New paragraph in Limitations subsection acknowledging edge-hardware deployment as future work.

Major Comment 10 — $\lambda = 0.5$ unjustified

> *Equation 8 uses $\lambda = 0.5$ to balance dataset proportion and inverse validation loss contributions to aggregation weights. No ablation or justification for this choice is provided.*

Response.

We added a λ-sensitivity ablation (new Table R-λ) across $\lambda \in \{0.00, 0.25, 0.50, 0.75, 1.00\}$ on all three datasets. The accuracy curve is concave with maximum at $\lambda = 0.5$ on every dataset. The endpoints ($\lambda = 0$: pure inverse-loss; $\lambda = 1$: pure dataset proportion / FedAvg-style) each lose ≈0.30% accuracy on average compared to the optimum, illustrating that both signals contribute. Importantly, the curve is flat within $\lambda \in [0.25, 0.75]$ — performance stays within 0.10% of the optimum — so the precise λ is non-critical and could be set anywhere in [0.4, 0.6] without re-tuning. This robustness is consistent with our motivation: $\lambda = 0.5$ represents an equal-weight prior in the absence of specific knowledge about client quality distributions; informative priors could shift λ but the gain is marginal.

Manuscript changes: New Table R-λ added. New paragraph in "FedMamba aggregation" subsection discussing the sensitivity and the robustness within [0.25, 0.75].

Reviewer #2

We thank Reviewer #2 for the constructive and balanced evaluation. Reviewer #2's five concerns substantially overlap with Reviewer #1's points; we address each below, with cross-references to the more detailed responses already given above.

Comment R2-1 — Number of repetitions and significance testing

> *Although experiments are repeated, the number of repetitions (three runs) may be relatively limited for high-stakes claims of superiority; additional statistical significance testing is not explicitly reported.*

Addressed in our response to Reviewer 1 Major 1. All experiments now use n = 10 seeds with paired t-tests, Wilcoxon signed-rank tests, Holm-Bonferroni correction, 95% confidence intervals, and Cohen's d effect sizes. See new Tables R1 and R-Sig.

Comment R2-2 — Real-world clinical deployment validation

> *Evaluation is restricted to publicly available benchmark datasets; real-world clinical deployment scenarios are not validated.*

We agree this is a real limitation, but it is beyond the scope of an algorithmic contribution. This paper proposes FedMamba-IoMT as a federated-learning framework and evaluates it on three large-scale benchmarks that collectively cover the IoMT threat surface: clinical IoT traffic (CICIoMT2024), industrial-edge IoT (Edge-IIoTset), and emulated multi-device IoT with per-device skew (Gotham 2025). End-to-end clinical-network validation requires ethics-board approval at participating institutions, regulatory clearance under the FDA's 2023 cybersecurity guidance for medical devices, and cross-site IT infrastructure — all of which constitute a separate deployment study and are not claimed here. The Limitations subsection has been extended with a paragraph stating this explicitly.

Comment R2-3 — τ = 0.8 sensitivity

> *The Byzantine defense mechanism uses a fixed threshold (τ = 0.8), and no sensitivity analysis for this parameter is provided, which may limit generalizability.*

Addressed in our response to Reviewer 1 Major 4 — full $\tau \in \{0.5, \ldots, 1.0\}$ sweep with FRR and TRR reporting at both $\alpha = 0.5$ and $\alpha = 0.1$, plus a theoretical sketch and adaptive-τ discussion. See new Table R-τ.

Comment R2-4 — Privacy leakage risks beyond gradient inversion

> *While privacy guarantees are formally defined, practical privacy leakage risks in real deployments are not deeply analyzed beyond gradient inversion experiments.*

We added a Privacy Threat Model subsection that discusses three additional leakage vectors: (a) membership inference attacks (we ran the Shokri et al. 2017 attack and report attacker AUC at each ε — ε = 3.0 reduces attacker AUC from 0.78 to 0.54, near chance); (b) attribute inference from the broadcast

global model (mitigated by the formal ε-DP guarantee composed across all rounds); (c) side-channel timing inference at the aggregator (orthogonal to FedMamba; secure aggregation protocols [Bonawitz et al. 2017] are a natural complement and are discussed as future integration work). A small new figure (Fig 18, membership-inference AUC vs. ε) accompanies this subsection.

Comment R2-5 — Hyperparameter selection and tokenization rationale

> *Some methodological choices (e.g., hyperparameter selection rationale, tokenization strategy justification) could be further elaborated for clarity.*

We have expanded the relevant discussion using ablations and figures already present in the manuscript:

- Hyperparameter choices: $T = 50$ rounds is justified by the convergence curves in Fig 2 — validation loss plateaus by round 30 on all three datasets, with $T = 50$ providing a safety margin. $E = 5$ local epochs balances communication efficiency against client compute; this is a conventional choice in cross-silo FL (consistent with FedAvg, FedProx, and FedIoMT). $\eta = 10^{-3}$ with ReduceLROnPlateau is a standard Adam configuration for tabular DL. Batch size 1024 is selected to keep all baselines comparable on A100 memory.
- Tokenization strategy: the mutual-information feature ordering is empirically validated in Fig 15 (feature-ordering ablation), which compares MI-ordered, random, alphabetical, and correlation-based orderings. The MI ordering provides the best accuracy across all three datasets. We have added a brief sentence in the "Feature tokenization" subsection noting the intuition: a higher-MI feature at the start of the pseudo-sequence provides a stronger conditioning signal for the Mamba selective scan, which is sensitive to early state initialization.

We have not added new appendix ablations beyond those already in the manuscript. The new tables introduced in this revision (R-FT, R-τ, R-DP, R-λ) directly address the more critical sensitivity concerns raised by Reviewer #1.

Closing

We are grateful for the depth and rigor of both reviews. The manuscript is substantially improved by the additional experiments, statistical analysis, and clarifications. We have implemented every requested change and have made supporting materials (code, configuration, per-seed logs) available at the repository linked in the Code Availability statement. We look forward to your further evaluation.

Respectfully,

Mohammed Tawfik, on behalf of all co-authors

Sana'a University, Faculty of Computer and Information Technology

m.tawfik@su.edu.ye | ORCID: 0000-0002-1227-387X
