# Supplementary material for: FedMamba-IoMT: Federated state space models with differential privacy and byzantine resilience for privacy-preserving intrusion detection in Internet of Medical Things
Source: PLoS One. 2026 Aug 10;21(8):e0355601. doi: 10.1371/journal.pone.0355601 (PMC13456290; doi:10.1371/journal.pone.0355601)
Supplement: Supplementary file 2 [file pone.0355601.s003.docx]

**Response to Academic Editor and Reviewers**

**Manuscript ID:** PONE-D-26-18683R1

**Title:** FedMamba-IoMT: Federated State Space Models with Differential Privacy and Byzantine Resilience for Privacy-Preserving Intrusion Detection in Internet of Medical Things

**Journal:** PLOS ONE

**Date:** July 22, 2026

Dear Dr. Saif,

We sincerely thank you and the reviewers for the careful evaluation of our revised manuscript and for the positive assessments received in this round. We are pleased that both reviewers consider all of their previous comments to have been adequately addressed. Below we respond to the editorial comment point by point and transparently document all changes made in this revision. All modifications are highlighted in the accompanying “Revised Manuscript with Track Changes” file.

**Response to the Academic Editor**

**Editor Comment 1:**

*“Please include a table presenting list of hyperparameter used in your algorithms and their respective values.”*

**Response:** We thank the editor for this valuable suggestion, which improves the reproducibility of our work. We have added a new consolidated table (Table 5 in the revised manuscript, referenced at the end of the “Data preprocessing and implementation details” subsection of Materials and Methods) that lists every hyperparameter used across all components of the FedMamba-IoMT framework together with its value, organized into seven groups: (i) the Mamba architecture (model dimension, SSM state dimension, convolution kernel, expansion factor, number of blocks, tokenization chunk size, dropout, classifier dimensions); (ii) the federated protocol (number of clients, communication rounds, local epochs, participation fraction, Dirichlet non-IID concentration, FedProx proximal coefficient); (iii) the FedMamba aggregation (mixing coefficient λ, numerical stabilizer); (iv) local optimization (optimizer, learning rate, batch size, learning-rate schedule, early stopping, weight initialization); (v) differential privacy (clipping norm, privacy budgets, failure probability, noise multipliers); (vi) the Byzantine defense (cosine-similarity threshold τ); and (vii) data preprocessing and experimental design (train/test split, SMOTE settings, and the ten random seeds). Where a hyperparameter was varied in a sensitivity study, the explored range is indicated alongside the chosen value.

**Response to Reviewer #2**

*“All comments have been addressed.”*

**Response:** We sincerely thank Dr. Muhammad Ali Abid for the constructive feedback provided throughout the review process, which has substantially improved the quality of the manuscript, and for the positive final assessment.

**Response to Reviewer #3**

*“Thanks to the author for their effort in improving the quality of the manuscript. The quality of the manuscript has been improved; no further comments.”*

**Response:** We sincerely thank Mr. Huang Yunfan for the thoughtful comments in the previous round and for the positive final assessment of the revised manuscript.

**Additional changes made in this revision**

While preparing this revision we performed a final internal-consistency audit of the manuscript. For full transparency, we document the resulting minor corrections below. None of these changes affects any experimental result, comparison, or conclusion of the paper.

- **Communication-cost accounting.** We detected an inconsistency between the stated per-round communication formula (Comm = 2·|S_t|·|θ|·b, with |S_t| = γK = 6 selected clients) and the absolute megabyte values previously reported. All per-round communication values now consistently follow the stated formula (e.g., FedMamba: 6.2 MB/round; FT-Transformer: 28.3 MB/round). Figures 14 and 18 were regenerated accordingly. All relative comparisons (e.g., the 78% communication reduction versus the Transformer baseline and the 83% reduction versus FedMedSecure) are unchanged, since every architecture is affected by the same constant factor.
- **Statistical reporting harmonization.** The ε = ∞ (no-DP) rows of the differential-privacy table now report exactly the same means and standard deviations as the overall performance table, removing a small residual inconsistency between the two tables.
- **Gotham 2025 taxonomy wording.** Because the 8 high-level Gotham classes include benign traffic, we now consistently describe them as “8 high-level traffic categories (benign plus seven attack behaviors)” throughout the abstract, contributions, dataset description, results, and conclusion, and corrected the corresponding row of the dataset summary table.
- **Comparison phrasing.** The sentence comparing our ε = 1.0 results with Torre et al. was reworded to make clear that the comparison concerns the strength of the privacy budget rather than a raw accuracy advantage at that operating point.

We thank the Academic Editor and the reviewers once again for their time and constructive guidance, and we hope the revised manuscript is now suitable for publication in PLOS ONE.

Sincerely,

Dr. Mohammed Tawfik (corresponding author, on behalf of all authors)

Faculty of Computer and Information Technology, Sana'a University, Sana'a, Yemen

Email: m.tawfik@su.edu.ye
