# Supplementary material for: FedMamba-IoMT: Federated state space models with differential privacy and byzantine resilience for privacy-preserving intrusion detection in Internet of Medical Things
Source: PLoS One. 2026 Aug 10;21(8):e0355601. doi: 10.1371/journal.pone.0355601 (PMC13456290; doi:10.1371/journal.pone.0355601)
Supplement: Peer review history

# Peer review: Decision Letter 0

**DOI:** 10.1371/journal.pone.0355601.r001

**Authors:** Sohail Saif

3 Jun 2026

FedMamba-IoMT: Federated State Space Models with Differential Privacy and Byzantine Resilience for Privacy-Preserving Intrusion Detection in Internet of Medical Things

PLOS One

Dear Dr. TAWFIK,

Thank you for submitting your manuscript to PLOS ONE. After careful consideration, we feel that it has merit but does not fully meet PLOS ONE’s publication criteria as it currently stands. Therefore, we invite you to submit a revised version of the manuscript that addresses the points raised during the review process.

Please submit your revised manuscript by Jul 18 2026 11:59PM. If you will need more time than this to complete your revisions, please reply to this message or contact the journal office at plosone@plos.org. When you're ready to submit your revision, log on to https://www.editorialmanager.com/pone/ and select the 'Submissions Needing Revision' folder to locate your manuscript file.

- A letter that responds to each point raised by the academic editor and reviewer(s). You should upload this letter as a separate file labeled 'Response to Reviewers'.
- A marked-up copy of your manuscript that highlights changes made to the original version. You should upload this as a separate file labeled 'Revised Manuscript with Track Changes'.
- An unmarked version of your revised paper without tracked changes. You should upload this as a separate file labeled 'Manuscript'.

If you would like to make changes to your financial disclosure, please include your updated statement in your cover letter. Guidelines for resubmitting your figure files are available below the reviewer comments at the end of this letter.

If applicable, we recommend that you deposit your laboratory protocols in protocols.io to enhance the reproducibility of your results. Protocols.io assigns your protocol its own identifier (DOI) so that it can be cited independently in the future. For instructions see: https://journals.plos.org/plosone/s/submission-guidelines#loc-laboratory-protocols. Additionally, PLOS ONE offers an option for publishing peer-reviewed Lab Protocol articles, which describe protocols hosted on protocols.io. Read more information on sharing protocols at https://plos.org/protocols?utm_medium=editorial-email&utm_source=authorletters&utm_campaign=protocols.

As the corresponding author, your ORCID iD is verified in the submission system and will appear in the published article. PLOS supports the use of ORCID, and we encourage all coauthors to register for an ORCID iD and use it as well. Please encourage your coauthors to verify their ORCID iD within the submission system before final acceptance, as unverified ORCID iDs will not appear in the published article. *Only*  the individual author can complete the verification step; PLOS staff *cannot*  verify ORCID iDs on behalf of authors.

We look forward to receiving your revised manuscript.

Kind regards,

Sohail Saif, Ph.D

Academic Editor

PLOS One

Journal Requirements:

When submitting your revision, we need you to address these additional requirements.

1. Please ensure that your manuscript meets PLOS ONE's style requirements, including those for file naming. The PLOS ONE style templates can be found at https://journals.plos.org/plosone/s/file?id=wjVg/PLOSOne_formatting_sample_main_body.pdf and https://journals.plos.org/plosone/s/file?id=ba62/PLOSOne_formatting_sample_title_authors_affiliations.pdf

2. Please note that PLOS One has specific guidelines on code sharing for submissions in which author-generated code underpins the findings in the manuscript. In these cases, we expect all author-generated code to be made available without restrictions upon publication of the work. Please review our guidelines at https://journals.plos.org/plosone/s/materials-and-software-sharing#loc-sharing-code and ensure that your code is shared in a way that follows best practice and facilitates reproducibility and reuse.

3. We note that Figure 1 in your submission contains images which may be copyrighted. All PLOS content is published under the Creative Commons Attribution License (CC BY 4.0), which means that the manuscript, images, and Supporting Information files will be freely available online, and any third party is permitted to access, download, copy, distribute, and use these materials in any way, even commercially, with proper attribution. For more information, see our copyright guidelines: http://journals.plos.org/plosone/s/licenses-and-copyright.

We require you to either (1) present written permission from the copyright holder to publish these figures specifically under the CC BY 4.0 license, or (2) remove the figures from your submission:

1. You may seek permission from the original copyright holder of Figure 1 to publish the content specifically under the CC BY 4.0 license.

We recommend that you contact the original copyright holder with the Content Permission Form (http://journals.plos.org/plosone/s/file?id=7c09/content-permission-form.pdf) and the following text:

“I request permission for the open-access journal PLOS ONE to publish XXX under the Creative Commons Attribution License (CCAL) CC BY 4.0 (http://creativecommons.org/licenses/by/4.0/). Please be aware that this license allows unrestricted use and distribution, even commercially, by third parties. Please reply and provide explicit written permission to publish XXX under a CC BY license and complete the attached form.”

Please upload the completed Content Permission Form or other proof of granted permissions as an "Other" file with your submission.

In the figure caption of the copyrighted figure, please include the following text: “Reprinted from [ref] under a CC BY license, with permission from [name of publisher], original copyright [original copyright year].”

2. If you are unable to obtain permission from the original copyright holder to publish these figures under the CC BY 4.0 license or if the copyright holder’s requirements are incompatible with the CC BY 4.0 license, please either i) remove the figure or ii) supply a replacement figure that complies with the CC BY 4.0 license. Please check copyright information on all replacement figures and update the figure caption with source information. If applicable, please specify in the figure caption text when a figure is similar but not identical to the original image and is therefore for illustrative purposes only.

4.  We note that while your manuscript text cites 18 main figures and have captions included, several of your uploaded figure items use descriptive text names rather than specific figure numbers.

To ensure that these figures files are correctly matched, please amend your submission and update your file names, with figure numbers indicated. Please ensure to renumber your files in a sequential order and update any in-text citations and provide figure captions for these files in your manuscript.

If the reviewer comments include a recommendation to cite specific previously published works, please review and evaluate these publications to determine whether they are relevant and should be cited. There is no requirement to cite these works unless the editor has indicated otherwise.

[Note: HTML markup is below. Please do not edit.]

Reviewers' comments:

Reviewer's Responses to Questions

**Comments to the Author**

1. Is the manuscript technically sound, and do the data support the conclusions?

Reviewer #1: Yes

Reviewer #2: Partly

**********

2. Has the statistical analysis been performed appropriately and rigorously?

Reviewer #1: No

Reviewer #2: Yes

**********

3. Have the authors made all data underlying the findings in their manuscript fully available?

The PLOS Data policy

Reviewer #1: Yes

Reviewer #2: Yes

**********

4. Is the manuscript presented in an intelligible fashion and written in standard English?

Reviewer #1: Yes

Reviewer #2: Yes

**********

Reviewer #1: This paper introduces FedMamba-IoMT, a federated learning framework for intrusion detection in Internet of Medical Things networks that adapts the Mamba selective State Space Model architecture to tabular network traffic data. The framework incorporates differential privacy via DP-SGD, a cosine similarity-based Byzantine resilience mechanism, and SHAP/LIME explainability. Experiments are conducted across three benchmark datasets Edge-IIoTset, CICIoMT2024, and Gotham Dataset 2025 reporting accuracy above 99% without differential privacy and above 97.8% at ε=3.0. The paper claims to be the first work combining Mamba with federated learning for intrusion detection.

The topic is timely and the engineering effort is substantial. However, several methodological concerns related to statistical validity, baseline definition, and the substantiation of key claims require resolution before the manuscript can be considered for publication.

Major Comments

1. All reported results are based on only three random seeds {42, 123, 456}. The primary claims of superiority over FedAvg and FedProx rest on accuracy margins of 0.36–0.64%, and the claims of state-of-the-art federated performance rest on margins of 0.08–0.10% standard deviation ranges. With only three experimental runs, these margins cannot be statistically distinguished from noise.

Specifically:

• No hypothesis tests (e.g., paired t-tests, Wilcoxon signed-rank tests) are reported for any pairwise comparison between FedMamba and baseline aggregation strategies.

• No confidence intervals are reported beyond ± standard deviation, which with n=3 yields extremely wide 95% confidence intervals that would likely overlap between methods.

• The claim that FedMamba "consistently outperforms" FedAvg and FedProx (Section: Federated aggregation ablation study) cannot be supported with three seeds and no significance testing.

Required action: The authors must either (a) increase the number of independent runs to a minimum of five, and preferably ten, and report statistical significance tests for all primary pairwise comparisons, or (b) provide a rigorous justification with power analysis for why three seeds are sufficient given the observed effect sizes. Simply reporting mean ± standard deviation across three seeds is not adequate for claims of this magnitude in a scientific publication.

2. The paper's central efficiency claims "78% fewer parameters than Transformer alternatives," "78% communication reduction," and "2× higher reconstruction error than Transformer-based FL" all rely on a "Transformer-IDS" with approximately 620K parameters that appears in Table 8 and throughout the results. However, this model is never defined.

There is no citation for this architecture, no description of the number of attention heads, layers, feedforward dimensions, or training configuration, and no indication of whether it was trained under the same federated protocol as FedMamba. The comparative accuracy of this Transformer baseline is also never reported, making it impossible to assess whether the efficiency gain comes at an accuracy cost.

This is a fundamental scientific transparency issue. Efficiency comparisons and privacy resistance comparisons against an undefined, uncitable baseline are not reproducible and cannot be peer-reviewed.

Required action: The authors must either (a) provide full architectural specifications and a citation or open-source implementation for the Transformer-IDS baseline, along with its federated accuracy under identical experimental conditions, or (b) replace this undefined baseline with a published, citable Transformer-based IDS model evaluated under identical conditions. All claims referencing "Transformer-IDS" must be substantiated against this properly defined baseline.

3. The authors identify FedIoMT (Fahim-Ul-Islam et al., Reference 36, IEEE Transactions on Consumer Electronics, 2025) as "the closest existing work to FedMamba-IoMT in concept," describing it as a federated learning framework with KAN and meta-learning for IoMT intrusion detection. Despite this acknowledgment, no direct quantitative comparison between FedMamba-IoMT and FedIoMT is provided on any shared dataset.

A paper claiming to advance the state of the art in federated IoMT intrusion detection cannot omit a direct comparison with its most closely related published competitor. The absence of this comparison is particularly notable given that the CICIoMT2024 dataset, which FedIoMT appears to evaluate on based on its publication venue and scope, is one of the three primary benchmarks in this paper.

Required action: The authors must include a direct experimental comparison against FedIoMT on at least one shared benchmark dataset, under equivalent or clearly described federated conditions. If the FedIoMT code or results are not reproducible, the authors must provide a detailed explanation and include whatever partial comparison is feasible, with an honest discussion of the limitations.

4. The cosine similarity threshold τ = 0.8 is a critical hyperparameter in the proposed Byzantine resilience mechanism. Updates below this threshold are rejected entirely from aggregation. The choice of τ directly governs the trade-off between filtering malicious updates and incorrectly rejecting legitimate updates from heterogeneous clients under non-IID conditions a particularly sensitive balance given that the paper simulates Dirichlet partitioning with α = 0.5, which produces substantial update direction variance even among honest clients.

The paper provides no theoretical justification for τ = 0.8, no empirical sensitivity analysis across alternative values, and no analysis of the false rejection rate (legitimate clients incorrectly filtered) at this threshold under varying degrees of data heterogeneity.

Required action: The authors must provide a sensitivity analysis of model performance across a range of τ values (e.g., τ ∈ {0.5, 0.6, 0.7, 0.8, 0.9}) under at least one dataset and α = 0.5. Additionally, the false rejection rate for honest clients must be reported at τ = 0.8. If the authors can derive a theoretical bound or heuristic for threshold selection based on the expected update distribution, this would substantially strengthen the contribution.

5. The paper claims formal (ε, δ)-differential privacy guarantees at five privacy budgets (ε ∈ {1.0, 2.0, 3.0, 5.0, 8.0}) with δ = 10^-5^ across T=50 rounds and E=5 local epochs. However, the noise multiplier σ the parameter that actually determines the privacy-utility trade-off and is required for independent verification of the claimed ε values — is never reported for any of the five privacy budgets.

Without knowing σ, C (gradient clipping norm, stated as 1.0), and the batch sampling ratio q for each dataset, it is impossible for reviewers or readers to verify that the claimed ε values are correctly computed. Furthermore, the paper states that ε = 3.0 is "a privacy budget widely considered suitable for medical applications," citing Abadi et al. (2016). This citation is to the original DP-SGD paper, which is a general machine learning privacy paper. There is no consensus standard for acceptable ε values in healthcare applications, and this citation misrepresents the state of the field.

Required action: (a) Report the noise multiplier σ for each ε budget in a table or appendix. (b) Report the batch sampling ratio q for each dataset, as this is a necessary input to the RDP composition. (c) Remove or substantially revise the claim that ε = 3.0 is "widely considered suitable for medical applications" and replace it with a more accurate characterization of the current state of the literature on privacy budgets in healthcare contexts.

6. The central architectural innovation is reformulating tabular network traffic features as pseudo-sequential tokens for processing by the Mamba selective scan mechanism. This is a non-trivial design choice because network traffic features (TCP flags, frame length, IP TTL, etc.) have no inherent sequential ordering or causal temporal structure in the tabular form used here.

The feature ordering ablation (Figure 15) shows that mutual information sorting yields approximately 0.3% improvement over random ordering. While the authors interpret this as validating their approach, an alternative interpretation is that the sequential processing provides only marginal benefit over a permutation-invariant architecture, and that a non-sequential model of similar parameter count might achieve comparable performance.

The paper does not compare FedMamba against any permutation-invariant tabular baseline such as a standard MLP, TabNet, or FT-Transformer with matched parameter counts under identical federated conditions. Without such a comparison, it cannot be determined whether the Mamba sequential processing is actually contributing to performance or whether the results are achievable with a simpler architecture.

Required action: The authors should include at least one comparison against a parameter-matched permutation-invariant baseline (e.g., a standard MLP with equivalent depth and width, or a simple attention-free model) under identical federated conditions on at least one dataset. This comparison is necessary to isolate the contribution of the sequential Mamba processing from the general benefits of the federated framework.

7. The paper presents reconstruction MSE and cosine similarity as evidence that FedMamba resists gradient inversion attacks, reporting that FedMamba provides "2× higher reconstruction error compared to Transformer-based FL" without differential privacy. However, the gradient inversion attack used for this evaluation is a self-implemented simulation against the authors' own undefined Transformer baseline. No published gradient inversion attack implementation (e.g., the original DLG implementation from Zhu et al. 2019, or the improved R-GAP or GradInversion attacks) is used.

Furthermore, the claim that compact parameterization inherently resists gradient inversion is theoretically reasonable but does not account for the fact that gradient inversion attack quality depends heavily on the specific attack algorithm, not just parameter count. Modern gradient inversion attacks for small models can be highly effective.

Required action: The authors should either (a) use a published, reproducible gradient inversion attack implementation for their experiments and clearly cite and describe the attack configuration, or (b) substantially soften their language regarding gradient inversion resistance, acknowledging that their MSE/cosine similarity metrics are proxies and not a demonstration of resistance against optimized attacks. The current presentation overstates the strength of this defense.

8. The Gotham Dataset 2025 is described as providing per-device non-IID traffic distributions from 78 emulated devices, making it "the first benchmark explicitly designed for federated learning IDS research." However, the experimental setup uses K=10 clients with Dirichlet partitioning, discarding the dataset's native per-device partitioning structure. The primary advantage of this dataset its ecologically valid, per-device federated structure is therefore not utilized. The authors should either use the native per-device partitioning for at least one experiment, or acknowledge that this advantage of the dataset is not exploited in the current work.

9. The 1.23ms inference latency is measured on an NVIDIA A100 GPU. IoMT edge gateways and medical device processors are typically ARM-based embedded systems with orders-of-magnitude less compute. The claim that FedMamba enables "real-time detection" based on A100 measurements is not transferable to the stated deployment context. The authors should either provide latency measurements on representative edge hardware, or qualify this claim explicitly as measured on server-class GPU hardware.

10. Equation 8 uses λ = 0.5 to balance dataset proportion and inverse validation loss contributions to aggregation weights. No ablation or justification for this choice is provided. A brief sensitivity analysis or theoretical rationale for this value would strengthen the aggregation contribution.

Reviewer #2: The manuscript presents a technically strong and timely contribution addressing privacy-preserving intrusion detection in IoMT using federated learning and State Space Models. The integration of Mamba architecture with differential privacy (DP-SGD) and Byzantine-resilient aggregation is novel and well-motivated (Introduction, pp. 2–3; Research gaps, p. 5). The experimental evaluation is comprehensive, covering three large-scale datasets with multiple classes and realistic non-IID settings (Table 3, p. 7), which strengthens the validity of the conclusions. The reported results (Tables 5–7, pp. 13–15) generally support the claims of high accuracy, efficiency, and robustness.

From a statistical and experimental standpoint, the study demonstrates good practices. Experiments are repeated three times with reported mean ± standard deviation (p. 12), and comparisons include relevant baselines such as FedAvg and FedProx (Table 6, p. 14). The use of multiple datasets with varying characteristics is a notable strength.

The manuscript is clearly written, logically structured, and uses appropriate academic language throughout. The problem formulation, methodology, and algorithmic details are well-described (pp. 9–12), making the work reproducible in principle.

However, several limitations should be noted:

Although experiments are repeated, the number of repetitions (three runs) may be relatively limited for high-stakes claims of superiority; additional statistical significance testing is not explicitly reported (Not clearly mentioned in the document).

Evaluation is restricted to publicly available benchmark datasets; real-world clinical deployment scenarios are not validated (Limitations, p. 19).

The Byzantine defense mechanism uses a fixed threshold (τ = 0.8), and no sensitivity analysis for this parameter is provided (p. 11), which may limit generalizability.

While privacy guarantees are formally defined, practical privacy leakage risks in real deployments are not deeply analyzed beyond gradient inversion experiments.

Some methodological choices (e.g., hyperparameter selection rationale, tokenization strategy justification) could be further elaborated for clarity.

Overall, the manuscript demonstrates solid technical merit and meaningful contributions, but certain aspects of validation, robustness analysis, and practical applicability could be strengthened to fully support its claims.

**********

what does this mean?). If published, this will include your full peer review and any attached files.

If you choose “no”, your identity will remain anonymous but your review may still be made public.

**Do you want your identity to be public for this peer review?**  For information about this choice, including consent withdrawal, please see our Privacy Policy

Reviewer #1: No

Reviewer #2: No

**********

[NOTE: If reviewer comments were submitted as an attachment file, they will be attached to this email and accessible via the submission site. Please log into your account, locate the manuscript record, and check for the action link "View Attachments". If this link does not appear, there are no attachment files.]

To ensure your figures meet our technical requirements, please review our figure guidelines: https://journals.plos.org/plosone/s/figures

You may also use PLOS’s free figure tool, NAAS, to help you prepare publication quality figures: https://journals.plos.org/plosone/s/figures#loc-tools-for-figure-preparation.

NAAS will assess whether your figures meet our technical requirements by comparing each figure against our figure specifications.

# Author comment: Author response to Decision Letter 1

**DOI:** 10.1371/journal.pone.0355601.r002

12 Jun 2026

test

Attachment Submitted filename: Response_to_Reviewers.pdf

# Peer review: Decision Letter 1

**DOI:** 10.1371/journal.pone.0355601.r003

**Authors:** Sohail Saif

22 Jul 2026

Dear Dr. TAWFIK,

Please submit your revised manuscript by Sep 05 2026 11:59PM. If you will need more time than this to complete your revisions, please reply to this message or contact the journal office at plosone@plos.org. When you're ready to submit your revision, log on to https://www.editorialmanager.com/pone/ and select the 'Submissions Needing Revision' folder to locate your manuscript file.

- A letter that responds to each point raised by the academic editor and reviewer(s). You should upload this letter as a separate file labeled 'Response to Reviewers'.
- A marked-up copy of your manuscript that highlights changes made to the original version. You should upload this as a separate file labeled 'Revised Manuscript with Track Changes'.
- An unmarked version of your revised paper without tracked changes. You should upload this as a separate file labeled 'Manuscript'.

If you would like to make changes to your financial disclosure, please include your updated statement in your cover letter. Guidelines for resubmitting your figure files are available below the reviewer comments at the end of this letter.

If applicable, we recommend that you deposit your laboratory protocols in protocols.io to enhance the reproducibility of your results. Protocols.io assigns your protocol its own identifier (DOI) so that it can be cited independently in the future. For instructions see: https://journals.plos.org/plosone/s/submission-guidelines#loc-laboratory-protocols. Additionally, PLOS ONE offers an option for publishing peer-reviewed Lab Protocol articles, which describe protocols hosted on protocols.io. Read more information on sharing protocols at https://plos.org/protocols?utm_medium=editorial-email&utm_source=authorletters&utm_campaign=protocols.

As the corresponding author, your ORCID iD is verified in the submission system and will appear in the published article. PLOS supports the use of ORCID, and we encourage all coauthors to register for an ORCID iD and use it as well. Please encourage your coauthors to verify their ORCID iD within the submission system before final acceptance, as unverified ORCID iDs will not appear in the published article. *Only*  the individual author can complete the verification step; PLOS staff *cannot*  verify ORCID iDs on behalf of authors.

We look forward to receiving your revised manuscript.

Kind regards,

Sohail Saif, Ph.D

Academic Editor

PLOS One

Journal Requirements:

If the reviewer comments include a recommendation to cite specific previously published works, please review and evaluate these publications to determine whether they are relevant and should be cited. There is no requirement to cite these works unless the editor has indicated otherwise.

Please review your reference list to ensure that it is complete and correct. If you have cited papers that have been retracted, please include the rationale for doing so in the manuscript text, or remove these references and replace them with relevant current references. Any changes to the reference list should be mentioned in the rebuttal letter that accompanies your revised manuscript. If you need to cite a retracted article, indicate the article’s retracted status in the References list and also include a citation and full reference for the retraction notice.

[Note: HTML markup is below. Please do not edit.]

Reviewers' comments:

Reviewer's Responses to Questions

**Comments to the Author**

Reviewer #2: All comments have been addressed

Reviewer #3: All comments have been addressed

**********

2. Is the manuscript technically sound, and do the data support the conclusions?

Reviewer #2: Yes

Reviewer #3: Yes

**********

3. Has the statistical analysis been performed appropriately and rigorously?

Reviewer #2: Yes

Reviewer #3: Yes

**********

4. Have the authors made all data underlying the findings in their manuscript fully available?

The PLOS Data policy

Reviewer #2: Yes

Reviewer #3: Yes

**********

5. Is the manuscript presented in an intelligible fashion and written in standard English?

Reviewer #2: Yes

Reviewer #3: Yes

**********

Reviewer #2: (No Response)

Reviewer #3: Thanks to the author for their effort in improving the quality of the manuscript. The quality of the manuscript has been improved; no further comments.

**********

what does this mean?). If published, this will include your full peer review and any attached files.

If you choose “no”, your identity will remain anonymous but your review may still be made public.

**Do you want your identity to be public for this peer review?**  For information about this choice, including consent withdrawal, please see our Privacy Policy

Reviewer #2: **Yes:** Dr Muhammad Ali Abid

Reviewer #3: **Yes:** Huang Yunfan

**********

[NOTE: If reviewer comments were submitted as an attachment file, they will be attached to this email and accessible via the submission site. Please log into your account, locate the manuscript record, and check for the action link "View Attachments". If this link does not appear, there are no attachment files.]

To ensure your figures meet our technical requirements, please review our figure guidelines: https://journals.plos.org/plosone/s/figures

You may also use PLOS’s free figure tool, NAAS, to help you prepare publication quality figures: https://journals.plos.org/plosone/s/figures#loc-tools-for-figure-preparation.

NAAS will assess whether your figures meet our technical requirements by comparing each figure against our figure specifications.

# Author comment: Author response to Decision Letter 2

**DOI:** 10.1371/journal.pone.0355601.r004

22 Jul 2026

Response to Academic Editor and Reviewers

Manuscript ID: PONE-D-26-18683R1

Title: FedMamba-IoMT: Federated State Space Models with Differential Privacy and Byzantine Resilience for Privacy-Preserving Intrusion Detection in Internet of Medical Things

Journal: PLOS ONE

Date: July 22, 2026

Dear Dr. Saif,

We sincerely thank you and the reviewers for the careful evaluation of our revised manuscript and for the positive assessments received in this round. We are pleased that both reviewers consider all of their previous comments to have been adequately addressed. Below we respond to the editorial comment point by point and transparently document all changes made in this revision. All modifications are highlighted in the accompanying “Revised Manuscript with Track Changes” file.

Response to the Academic Editor

Editor Comment 1:

“Please include a table presenting list of hyperparameter used in your algorithms and their respective values.”

Response: We thank the editor for this valuable suggestion, which improves the reproducibility of our work. We have added a new consolidated table (Table 5 in the revised manuscript, referenced at the end of the “Data preprocessing and implementation details” subsection of Materials and Methods) that lists every hyperparameter used across all components of the FedMamba-IoMT framework together with its value, organized into seven groups: (i) the Mamba architecture (model dimension, SSM state dimension, convolution kernel, expansion factor, number of blocks, tokenization chunk size, dropout, classifier dimensions); (ii) the federated protocol (number of clients, communication rounds, local epochs, participation fraction, Dirichlet non-IID concentration, FedProx proximal coefficient); (iii) the FedMamba aggregation (mixing coefficient λ, numerical stabilizer); (iv) local optimization (optimizer, learning rate, batch size, learning-rate schedule, early stopping, weight initialization); (v) differential privacy (clipping norm, privacy budgets, failure probability, noise multipliers); (vi) the Byzantine defense (cosine-similarity threshold τ); and (vii) data preprocessing and experimental design (train/test split, SMOTE settings, and the ten random seeds). Where a hyperparameter was varied in a sensitivity study, the explored range is indicated alongside the chosen value.

Response to Reviewer #2

“All comments have been addressed.”

Response: We sincerely thank Dr. Muhammad Ali Abid for the constructive feedback provided throughout the review process, which has substantially improved the quality of the manuscript, and for the positive final assessment.

Response to Reviewer #3

“Thanks to the author for their effort in improving the quality of the manuscript. The quality of the manuscript has been improved; no further comments.”

Response: We sincerely thank Mr. Huang Yunfan for the thoughtful comments in the previous round and for the positive final assessment of the revised manuscript.

Additional changes made in this revision

While preparing this revision we performed a final internal-consistency audit of the manuscript. For full transparency, we document the resulting minor corrections below. None of these changes affects any experimental result, comparison, or conclusion of the paper.

• Communication-cost accounting. We detected an inconsistency between the stated per-round communication formula (Comm = 2·|S_t|·|θ|·b, with |S_t| = γK = 6 selected clients) and the absolute megabyte values previously reported. All per-round communication values now consistently follow the stated formula (e.g., FedMamba: 6.2 MB/round; FT-Transformer: 28.3 MB/round). Figures 14 and 18 were regenerated accordingly. All relative comparisons (e.g., the 78% communication reduction versus the Transformer baseline and the 83% reduction versus FedMedSecure) are unchanged, since every architecture is affected by the same constant factor.

• Statistical reporting harmonization. The ε = ∞ (no-DP) rows of the differential-privacy table now report exactly the same means and standard deviations as the overall performance table, removing a small residual inconsistency between the two tables.

• Gotham 2025 taxonomy wording. Because the 8 high-level Gotham classes include benign traffic, we now consistently describe them as “8 high-level traffic categories (benign plus seven attack behaviors)” throughout the abstract, contributions, dataset description, results, and conclusion, and corrected the corresponding row of the dataset summary table.

• Comparison phrasing. The sentence comparing our ε = 1.0 results with Torre et al. was reworded to make clear that the comparison concerns the strength of the privacy budget rather than a raw accuracy advantage at that operating point.

We thank the Academic Editor and the reviewers once again for their time and constructive guidance, and we hope the revised manuscript is now suitable for publication in PLOS ONE.

Sincerely,

Dr. Mohammed Tawfik (corresponding author, on behalf of all authors)

Faculty of Computer and Information Technology, Sana'a University, Sana'a, Yemen

Email: m.tawfik@su.edu.ye

Attachment Submitted filename: Response_to_Reviewers.docx

# Editor report: Decision Letter 2

**DOI:** 10.1371/journal.pone.0355601.r005

**Authors:** Sohail Saif

23 Jul 2026

FedMamba-IoMT: Federated State Space Models with Differential Privacy and Byzantine Resilience for Privacy-Preserving Intrusion Detection in Internet of Medical Things

PONE-D-26-18683R2

Dear Dr. TAWFIK,

We’re pleased to inform you that your manuscript has been judged scientifically suitable for publication and will be formally accepted for publication once it meets all outstanding technical requirements.

Within one week, you’ll receive an e-mail detailing the required amendments. When these have been addressed, you’ll receive a formal acceptance letter and your manuscript will be scheduled for publication.

An invoice will be generated when your article is formally accepted. Please note, if your institution has a publishing partnership with PLOS and your article meets the relevant criteria, all or part of your publication costs will be covered. Please make sure your user information is up-to-date by logging into Editorial Manager at Editorial Manager® and clicking the ‘Update My Information' link at the top of the page. For questions related to billing, please contact billing support.

If your institution or institutions have a press office, please notify them about your upcoming paper to help maximize its impact. If they’ll be preparing press materials, please inform our press team as soon as possible -- no later than 48 hours after receiving the formal acceptance. Your manuscript will remain under strict press embargo until 2 pm Eastern Time on the date of publication. For more information, please contact onepress@plos.org.

Kind regards,

Sohail Saif, Ph.D

Academic Editor

PLOS One

Additional Editor Comments (optional):

Revision is satisfactory.

Reviewers' comments:

# Editor report: Acceptance letter

**DOI:** 10.1371/journal.pone.0355601.r006

**Authors:** Sohail Saif

PONE-D-26-18683R2

PLOS One

Dear Dr. Tawfik,

I'm pleased to inform you that your manuscript has been deemed suitable for publication in PLOS One. Congratulations! Your manuscript is now being handed over to our production team.

At this stage, our production department will prepare your paper for publication. This includes ensuring the following:

* All references, tables, and figures are properly cited

* All relevant supporting information is included in the manuscript submission,

* There are no issues that prevent the paper from being properly typeset

You will receive further instructions from the production team, including instructions on how to review your proof when it is ready. Please keep in mind that we are working through a large volume of accepted articles, so please give us a few days to review your paper and let you know the next and final steps.

Lastly, if your institution or institutions have a press office, please let them know about your upcoming paper now to help maximize its impact. If they'll be preparing press materials, please inform our press team within the next 48 hours. Your manuscript will remain under strict press embargo until 2 pm Eastern Time on the date of publication. For more information, please contact onepress@plos.org.

You will receive an invoice from PLOS for your publication fee after your manuscript has reached the completed accept phase. If you receive an email requesting payment before acceptance or for any other service, this may be a phishing scheme. Learn how to identify phishing emails and protect your accounts at https://explore.plos.org/phishing.

If we can help with anything else, please email us at customercare@plos.org.

Thank you for submitting your work to PLOS ONE and supporting open access.

Kind regards,

PLOS ONE Editorial Office Staff

on behalf of

Dr. Sohail Saif

Academic Editor

PLOS One
